# Development of the titanium–TADDOLate-catalyzed asymmetric fluorination of β-ketoesters

**DOI:** 10.3762/bjoc.7.166

**Published:** 2011-10-17

**Authors:** Lukas Hintermann, Mauro Perseghini, Antonio Togni

**Affiliations:** 1 Laboratorium für Anorganische Chemie, ETH Zürich, Wolfgang-Pauli-Str. 10, 8093 Zürich, Switzerland; 2 Department Chemie, Technische Universität München, Lichtenbergstr. 4, 85748 Garching bei München, Germany

**Keywords:** Asymmetric catalysis, fluorination, fluoroorganic compounds, TADDOL, titanium

## Abstract

Titanium-based Lewis acids catalyze the α-fluorination of β-ketoesters by electrophilic N–F-fluorinating reagents. Asymmetric catalysis with TADDOLato–titanium(IV) dichloride (TADDOL = α,α,α',α'-tetraaryl-(1,3-dioxolane-4,5-diyl)-dimethanol) Lewis acids produces enantiomerically enriched α-fluorinated β-ketoesters in up to 91% enantiomeric excess, with either F–TEDA (1-chloromethyl-4-fluoro-1,4-diazoniabicyclo[2.2.2]octane bis(tetrafluoroborate)) in acetonitrile solution or NFSI (*N*-fluorobenzenesulfonimide) in dichloromethane solution as fluorinating reagents. The effects of various reaction parameters and of the TADDOL ligand structure on the catalytic activity and enantioselectivity were investigated. The absolute configuration of several fluorination products was assigned through correlation. Evidence for ionization of the catalyst complex by chloride dissociation, followed by generation of titanium β-ketoenolates as key reaction intermediates, was obtained. Based on the experimental findings, a general mechanistic sketch and a steric model of induction are proposed.

## Introduction

**Figure 1:**
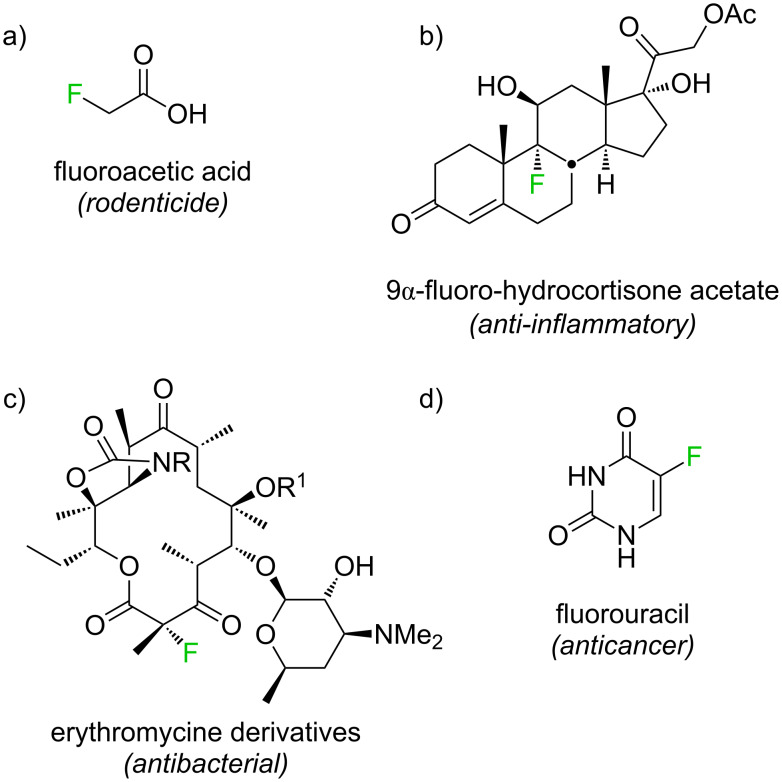
Fluorinated substances of biomedical relevance.

Fluoroorganic compounds have peculiar properties, rendering them interesting for a variety of specific applications [1–13]. Single substitutions of hydrogen by fluorine can change the biological effects of low-molecular-weight organic compounds tremendously, for example, unlike acetic acid, fluoroacetic acid (Figure 1a) is highly toxic [14]. Modulation of drug action by inclusion of fluorine, described already in 1954 for steroids (Figure 1b) [15], is now regularly explored in medicinal chemistry. An example of interest for the present work is the modification of the antibiotic erythromycine by α-fluorination of a β-ketoester substructure (Figure 1c) [16]. Fluorinated agrochemicals and drugs are now produced industrially on a large scale by a range of methods, including reactions with notoriously reactive fluorine gas in the production of the anticancer drug fluorouracil (Figure 1d) [17].

**Scheme 1:**
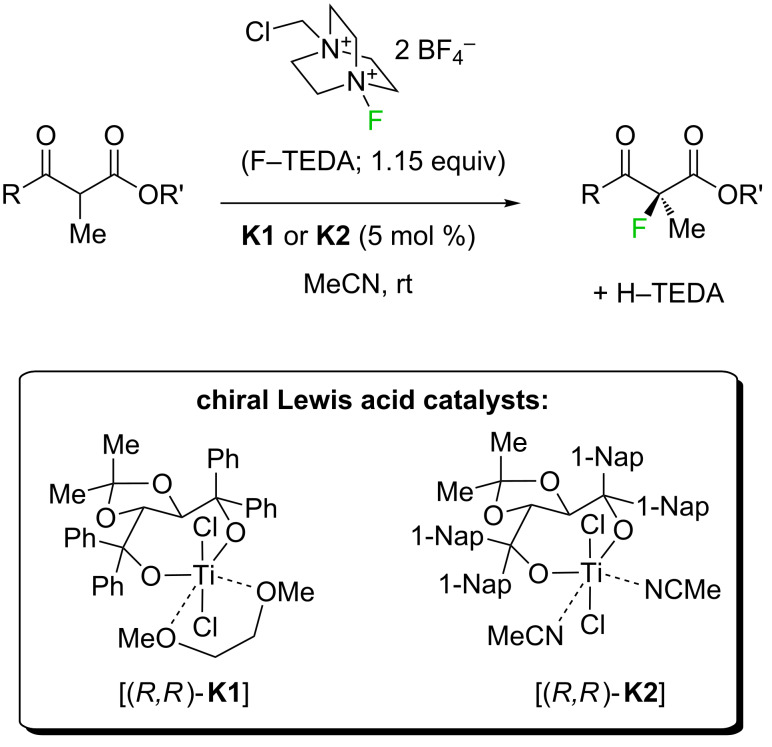
Enantioselective electrophilic fluorination catalyzed by TADDOLates **K1**, **K2**. TADDOL = α,α,α',α'-tetraaryl-(2,2-dimethyl-1,3-dioxolane-4,5-diyl)dimethanol. Phenyl-TADDOL (**T1**) = tetraphenyl-(2,2-dimethyl-1,3-dioxolane) derivative; 1-naphthyl-TADDOL (**T2**) = tetra-1-naphthyl-(2,2-dimethyl-1,3-dioxolane) derivative.

However, many specifically fluorinated compounds remain difficult to access because of limitations of the synthetic methodology or unpredictable reaction outcomes in the case of fluorinated starting materials. Seebach coined the term “flustrates” for “fluorinated substrates”; a term that also hints at the frustrations experienced by synthetic chemists in their struggle to prepare a desired fluoroorganic target [18]. A major synthetic challenge of fluoroorganic chemistry is the enantioselective generation of fluorinated stereogenic carbon centers [2,19–20]. Initially, chiral auxiliary approaches and diastereoselective reactions were developed, before Differding and Lang found the first stoichiometric asymmetric fluorination of β-ketoester enolates with a chiral N–F (*N*-fluoroamine) reagent in 1988 [21]. Later work by Davis [22–23], Takeuchi [24] and their respective coworkers extended this chemistry, while Haufe and coworkers were able to open *meso*-epoxides asymmetrically with HF equivalents and chiral chromium–salen complexes [25–26]. In the year 2000, two conceptually different applications of Banks’ electrophilic fluorinating reagent F–TEDA (1-chloromethyl-4-fluoro-1,4-diazoniabicyclo[2.2.2]octane bis(tetrafluoroborate); TEDA = triethylenediamine) [27–29] marked some important discoveries: First, a new generation of highly enantioselective chiral fluorinating reagents, derived by fluorine transfer [30] from F–TEDA to the quinuclidine portion of cinchona-alkaloids, was introduced by the groups of Cahard [31–33] and Shibata [34–35]. Second, the research efforts of our group towards realizing metal-catalyzed fluorinations [36–38] and asymmetric catalytic fluorination reactions [39], successfully channeled into the discovery of a catalytic asymmetric α-fluorination of β-ketoesters (Scheme 1) by means of the reagent F–TEDA and chiral titanium Lewis acid catalysts of the TiCl_2_(TADDOLate) type [40–42]. The same catalytic reaction principle has also allowed the performance of asymmetric chlorinations and brominations of β-ketoesters [43–45].

After the initial report [40], many metal-catalyzed asymmetric fluorinations were discovered by others and ourselves, based on complexes of Pd(II) [46], Cu(II) [47–49], Ni(II) [48,50] and Ru(II) [51] as catalysts [4,51]. In parallel, organocatalytic asymmetric electrophilic fluorination of carbonyl compounds by means of phase-transfer catalysts [52] and small-molecule chiral amine catalysts were introduced and explored with great success [53–56]. Here we present the full range of observations on titanium-catalyzed fluorinations of β-ketoesters. The focus is on the development of the reaction and the study of factors influencing its stereoselectivity depending on reaction parameters and catalyst-ligand effects. We also present stereochemical correlations, to assign the absolute configuration of the fluorination products, and observations relevant to the mechanism of the catalytic reaction. A subsequent paper will cover the substrate range of the catalytic fluorination and its extension to other activated carbonyl compounds [57].

## Results and Discussion

### Lewis acid catalyzed halogenation of activated carbonyl compounds

#### Overview

**Scheme 2:**
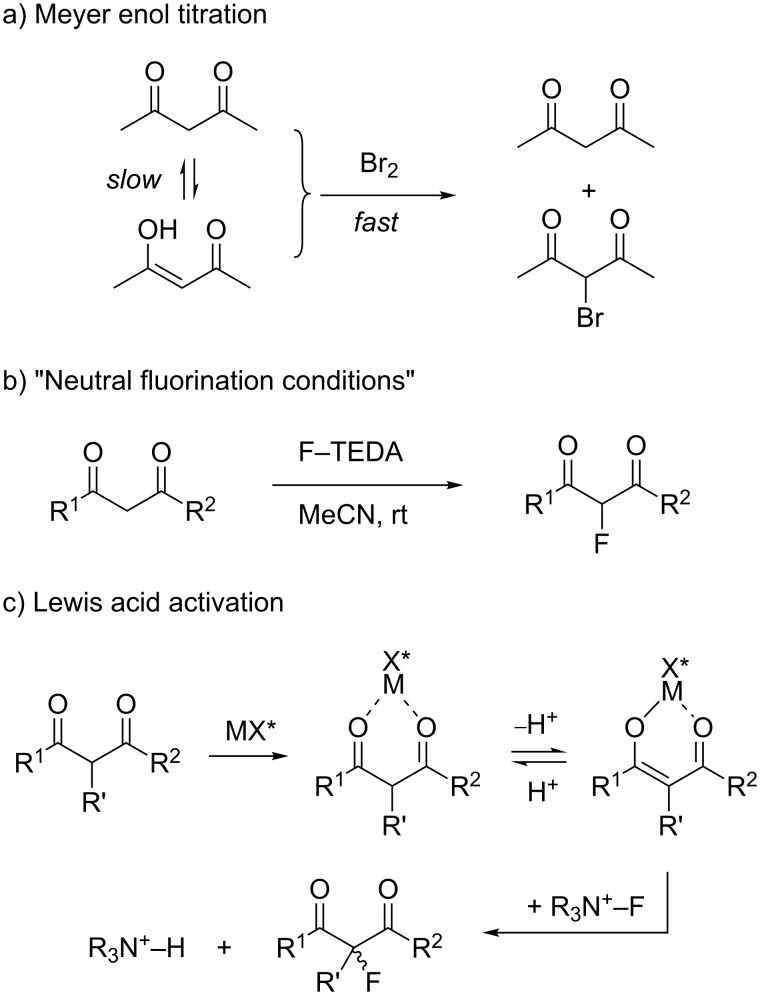
Halogenation of β-ketocarbonyl compounds: Importance of enolization and the potential role of a metal catalyst.

Nonfunctionalized ketones are halogenated in the α-position by halogens and other electrophilic halogenating agents [58–62]. These reactions can be slow due to rate-limiting enolization of the substrate, thus enolates or enolate equivalents are often preferred as substrates [58]. If activated methylene derivatives are mixed with a strong halogenating agent, the enol portion of the tautomeric equilibirum mixture is halogenated quickly, leaving behind the keto tautomer [63]. Further halogenation is then limited by the rate of enolization. This effect forms the basis of Meyer’s famous enol-titration experiments for determining the degree of enolization in activated carbonyl compounds (Scheme 2a) [63–65]. Preparative halogenations of 1,3-dicarbonyl substrates at ambient temperatures are relatively slow and it is more common to halogenate their alkali enolates instead [58]. From studies on the electrophilic fluorination with N–F reagents, it is known that some β-carbonyl compounds are α-fluorinated by simple combination of the reactants in solution at room temperature (Scheme 2b) [66–71]. The ease of this “neutral fluorination” protocol is directly connected to the ability of a substrate to spontaneously enolize. We hypothesized that catalytic acceleration of the enolization should also result in catalysis of the overall electrophilic halogenation. As a working hypothesis, it was assumed that coordination of β-ketocarbonyl compounds to Lewis acidic metal centers should facilitate their transformation to a covalently coordinated enolate, which may be fluorinated at carbon with asymmetric induction exerted by chiral steering ligands X* at the metal (Scheme 2c).

Incidentally, Meyer had already observed acceleration of the keto-enol equilibration of acetoacetic ester in the presence of iron(III) chloride in ethanol solution [64]. Scattered reports on polyhalogenations of ketones in the presence of antimony chloride have appeared [59], while Umemoto used Lewis acids in substoichiometric amounts (0.4 equiv of ZnCl_2_ or AlCl_3_) to accelerate fluorinations of 1,3-dicarbonyl compounds with *N*-fluoropyridinium salts [72]. In 1998, Chambers and Hutchinson reported the reaction of malonic esters with elemental fluorine in the presence of hydrated copper(II) nitrate at the 10 mol % level [73]. Still, the generality and synthetic potential of Lewis acid catalyzed α-halogenations of carbonyl compounds were not established.

#### Reactivity screening: Fluorination of β-ketoesters in the presence of Lewis acids

**Figure 2:**
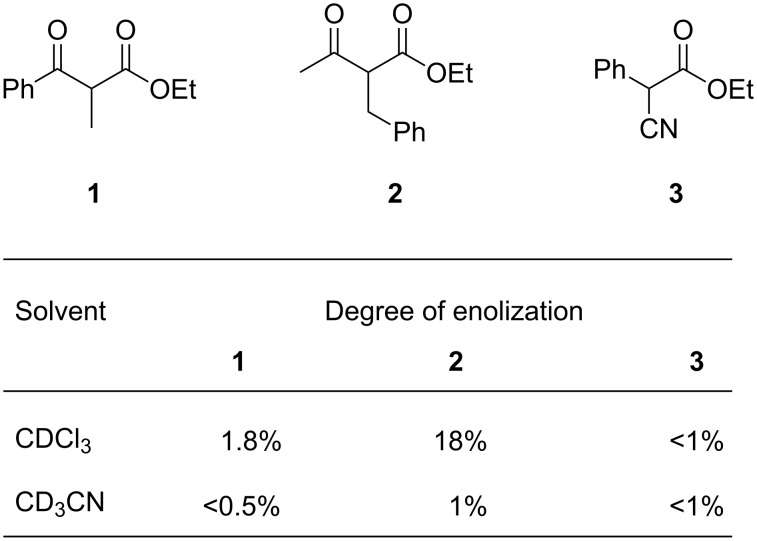
Model substrates for catalytic fluorinations, with the degree of enolization determined by ^1^H NMR measurements.

As a starting point for our investigation, we chose to study the fluorinations of the readily enolizable β-ketoesters **1** and **2** and of α-cyanoester **3** as model compounds with the reagent F–TEDA under either “neutral” conditions [66–71] or in the presence of Lewis acids (Figure 2).

**Figure 3:**
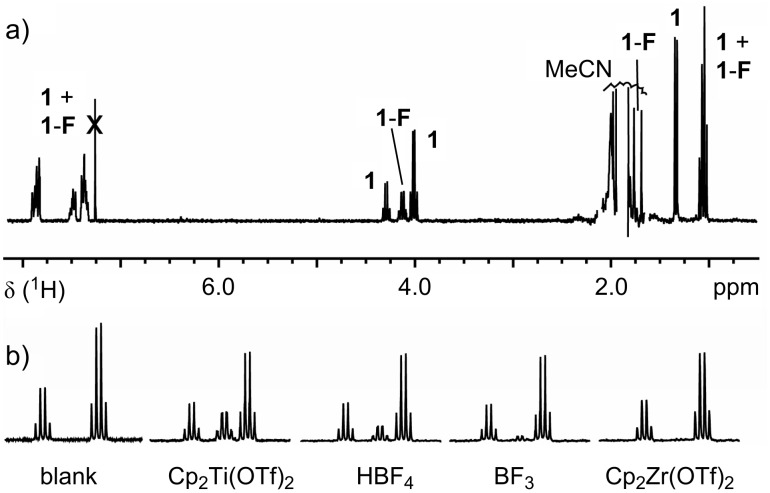
^1^H NMR (250 MHz) spectra of fluorination reaction mixtures diluted with CDCl_3_ and filtered. a) Full range spectrum with signals from substrate **1** and product **1**-**F**. The catalyst was Cp_2_Ti(OTf)_2_. Signals resulting from F–TEDA or H–TEDA are missing because of the insolubility of those species in CDCl_3_. The signals for MeCN and its ^13^C-satellites have been cut for clarity. b) Reaction progress monitoring after 28 h (region 3.9–4.3 ppm) in catalytic reactions with the Lewis acids indicated, along with the results from a blank experiment.

Freshly dissolved ethyl benzoylpropionate **1** contained some enol tautomer in CDCl_3_. In CD_3_CN, the value decreased to below 0.5% on standing (Figure 2). Fluorination of **1** in a saturated solution of F–TEDA in acetonitrile was negligible (<1%) after two weeks at ambient temperature, assuring us that a background reaction would not interfere when searching for catalytic reactions. After the addition of 5 to 10 mol % of Lewis acids to solutions of **1** and F–TEDA in acetonitrile, the reaction course (**1**→**1-F**) was monitored by ^1^H NMR analysis. Figure 3 shows representative ^1^H NMR spectra of reaction samples removed after 28 h reaction time at ambient temperature. The catalysts and the corresponding conversions induced by them for the fluorination of substrates **1** and **2** are listed in Table 1.

**Table 1:** Fluorination of **1** and **2** in the presence of different Lewis acids.

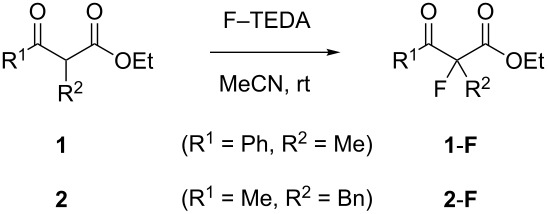				

Entry	Catalyst (mol %)	Conversion after 28 h (%)		
		**1**→**1**-**F**	**2**→**2**-**F**	
			neat^a^	equilibrated^b^

1	none (“blank”)	0	20	5
2	Cp_2_Ti(OTf)_2_ (5)	38	>95	95
3	Cp_2_Zr(OTf)_2_(thf) (5)	≤2	n.d.^c^	8
4	HBF_4_·H_2_O (10)	24	47	47
5	BF_3_·Et_2_O (10)	11	31	22
6	ZnF_2_ (5)	n.d.	25	n.d.

^a^neat = substrate added in substance. ^b^equilibrated = substrate added as stock solution in MeCN. ^c^n.d. = not determined.

**Scheme 3:**
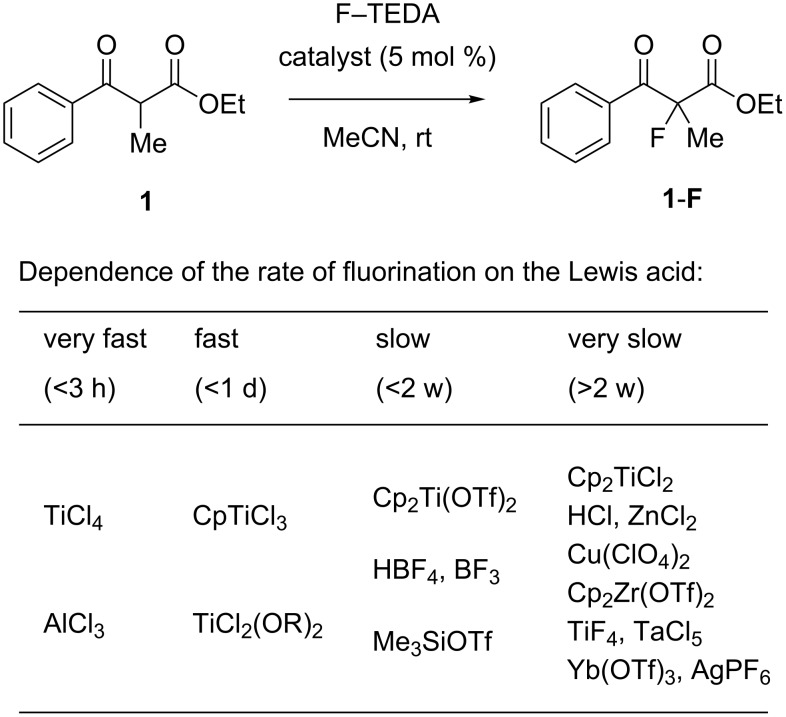
Qualitative ordering of catalytic activity of several Lewis acids in the fluorination **1**→**1**-**F**.

The titanium complex Cp_2_Ti(OTf)_2_ (Cp = *η*^5^-cyclopentadienyl) catalyzed the fluorination of both **1** and **2** (Table 1, entry 2), whereas the corresponding zirconium complex was inactive (Table 1, entry 3). Both the Brønsted acid HBF_4_ and the Lewis acid BF_3_ accelerated the reaction. Substrate **2** with a higher tendency towards enolization (Figure 2) gave higher conversions, but a similar trend. Interestingly, the conversions were different when substrate **2** was added to the reaction mixture in neat form compared to when it was added as a stock solution in MeCN (Table 1). This appears to reflect the enol content of **2** (Figure 2), which is much higher in a freshly dissolved sample than in an aged solution of a polar solvent. Presumably, the enol tautomer reacts uncatalyzed with F–TEDA, followed by a slow, Lewis acid catalyzed fluorination of the remaining ketone. The catalytic effects of other Lewis acids in the fluorination of **1** were semiquantitatively studied by TLC experiments and found as presented in Scheme 3.

Strong catalytic activity was observed for complexes of titanium and aluminum. Activity increases with the number of strongly σ-acidic ligands (TiCl_4_ > CpTiCl_3_ > Cp_2_Ti(OTf)_2_) and with the σ-acidity of the ligands themselves (Cp_2_Ti(OTf)_2_ > Cp_2_TiCl_2_). Fluorides as strong π-donors are not suitable ligands for Lewis acidic centers in the present reaction, as implied by the inactivity of TiF_4_. Organometallic Cp-complexes (Cp = η^5^-cyclopentadienyl) are suitable halogenation catalysts, even though halogenolysis of the metal–carbon bonds might have been expected [27]. For metals other than Ti and Al, no significant catalytic activity was observed in this specific model reaction (Scheme 3). The fluorination of cyanoester **3** with F–TEDA was not accelerated by any of the potential catalysts investigated (TiCl_4_, BF_3_·Et_2_O, SnCl_4_, Sn(OTf)_2_, AlCl_3_, Mg(OTf)_2_, ZnF_2_ or SbF_3_). The conversion **3**→**3**-**F** in a blank reaction amounted to 20% (3 d, MeCN, rt) or 40% (4.5 h, MeCN, 40 °C), but the addition of any of the aforementioned substances rather suppressed the reaction. This may be explained by the inability of **3** to bind in a chelating mode to metal complexes. In fact, when ketoesters **1** or **2** were mixed with TiCl_4_ in MeCN, they produced yellow to red colors characteristic for β-dicarbonylate complexes, whereas solutions of **3** remained colorless.

#### Catalytic halogenation under “neutral” conditions

**Scheme 4:**
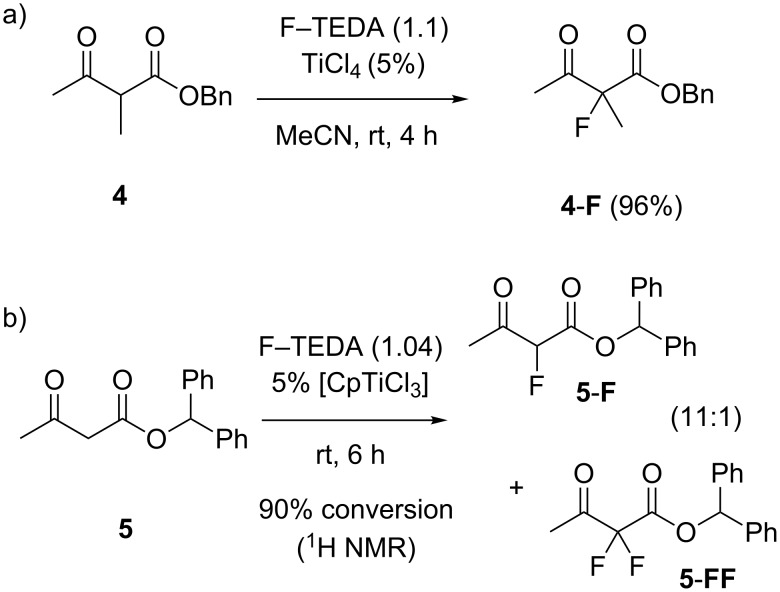
Catalysis of the “neutral” fluorination of β-ketoesters with F–TEDA by Lewis acidic titanium complexes.

While the remainder of this paper is concerned with the development of asymmetric catalytic halogenations, the Lewis acid catalyzed fluorination under neutral conditions (i.e., without stoichiometric base) is an attractive method also for carrying out nonstereoselective halogenations, which can profit from catalyst optimization for controlling reaction selectivity. Reactions were readily performed on preparative scale as shown for α-methyl-β-ketoester **4**, which was fluorinated in high yield with the aid of TiCl_4_ as a catalyst (Scheme 4a). With the same catalyst, β-ketoester **5** suffered partial cleavage of the ester group, but the milder Lewis acid CpTiCl_3_ induced a clean fluorination with high selectivity towards monofluorination (Scheme 4b) [74–75]. We have further explored aspects of selective monofluorination and sequential mixed dihalogenation elsewhere [74]. This example illustrates the potential of catalyst tuning for specific substrates in catalytic fluorination reactions. TiCl_4_ as well as CpTiCl_3_ (and other Lewis acids) [76–78] also catalyzed the chlorination of β-ketoesters with *N*-chlorosuccinimide or *N*-chlorosulfonamides as halogenating agents [43]. The Lewis acid catalyzed halogenations of β-ketoesters present an interesting alternative to either radical or neutral noncatalyzed reaction modes.

#### Catalytic asymmetric fluorination

**Figure 4:**
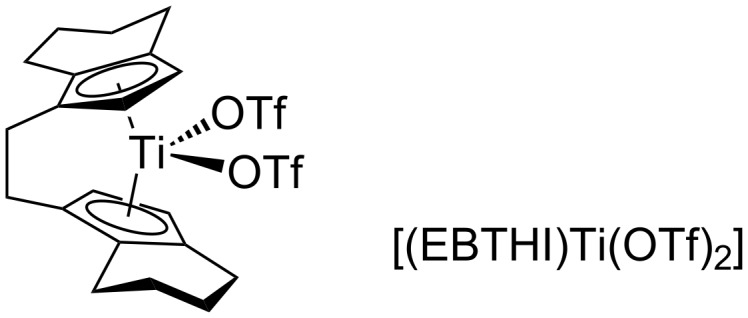
Structure of the chiral ansa-metallocene [(EBTHI)Ti(OTf)_2_].

Having shown the viability of Lewis acid catalyzed fluorination, we focused on the development of an asymmetric catalytic fluorination reaction using enantiopure Lewis acid catalysts. Based on the screening results (Scheme 3), titanium or aluminum catalysts were logical choices. An enantiopure sample of the ansa-titanocene [(EBTHI)Ti(OTf)_2_] (Figure 4) [79–81] indeed catalyzed the slow fluorination of **1** (14% conversion after 18 h, 25% after 36 h, 30 d to completion) or **2** (85% conversion after 18 h), but gave racemic products **1**-**F** and **2**-**F**.

Dialkoxyaluminium chlorides, which were prepared in situ from Me_2_AlCl and several chiral diols, were not catalytically active. However, the well-established Lewis acid [(*R*,*R*-TADDOLato)TiCl_2_] [82] (Scheme 1) displayed good catalytic activity (5 mol %, **1→1**-**F** in 4 h) and gave (*S*)-**1**-**F** with an enantiomeric excess of 28% (for a complex incorporating the ligand (*R,R*)-phenyl-TADDOL (**L1**)) (Table 2). Consistent catalysis results were obtained when the crystalline Lewis acid complexes **K1** and **K2** were applied instead of the complexes generated in situ. The synthesis, characterization and X-ray structures of these complexes, which are now commercially available, have been described elsewhere [83].

**Table 2:** Effect of catalyst loading, temperature, and water content on the fluorination of **1** with catalyst **K1**.^a^

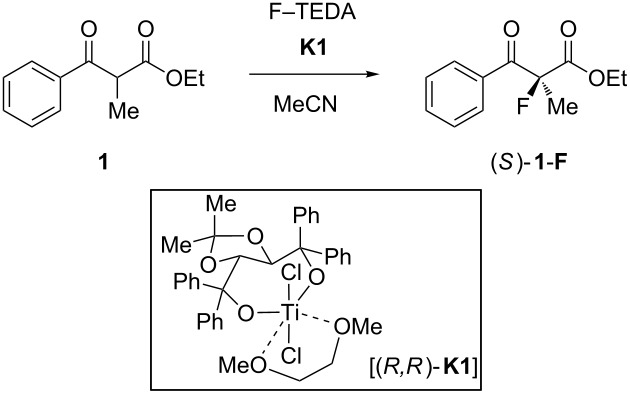					

Entry	**K1** (mol %)	*T* (°C)	Additives (mol %)	*t* (h)	ee (%)

1	1.2	rt		45^b^	25.2
2	5	rt		6	26.6
3	10	rt		2	26.5
4	20	rt		<2	25.5
5	50	rt		<0.3	23.4
6	100	rt		<0.3	22.4
7	5	rt	MS 3 Å	6	27
8	5	rt		6	28.5^c^
9	5	rt	H_2_O (5)	10	28.5
10	5	rt	H_2_O (50)	>12^d^	28
11	5	rt	H_2_O (1000)	–	–^e^
12	5	0		16	32
13	5	rt		<8	28
14	5	40		<2	23
15	5	60		0.1^f^	18

^a^All reactions were performed in reagent grade acetonitrile stored over 3 Å molecular sieves and run to complete conversion (TLC), unless otherwise mentioned. ^b^Reaction stopped before complete conversion. ^c^Entries 1–6 vs 7–11 vs 12–15 were run with different batches of F–TEDA, solvent and catalyst, leading to small selectivity deviations under seemingly identical conditions. ^d^50% conversion. ^e^No reaction. ^f^Conversion stopped at 80%.

#### Effects of catalyst loading, moisture, and temperature

The reaction rate of catalytic fluorination increased with the catalyst loading, but there was little change in the enantioselectivity (Table 2, entries 1–6). A minor decrease of selectivity at higher catalyst loadings is attributed to the temperature effect of the fast exothermic reactions. At low catalyst loadings (<2 mol %) catalyst poisoning occurs, presumably, by moisture and/or fluoride impurities from the reagent F–TEDA. The negative effect of fluoride anions on the catalytic activity is suggested by the inactivity of TiF_4_ as opposed to TiCl_4_ (Scheme 3), and by the finding that an in situ catalyst prepared from Ti(OiPr)_4_, TiF_4_ and TADDOL (1:1:2) (in situ [TiF_2_(TADDOLate)], but not characterized) was inactive. The controlled addition of water to catalytic runs did not affect the selectivity, but the activity decreased at high moisture levels (Table 2, entries 7–11). Use of reagent-grade acetonitrile, stored over molecular sieves (3 Å) in air, was satisfactory for most purposes. Performing the reaction in the presence of powdered molecular sieves had no beneficial effects (Table 2, entry 7). All catalytic runs were conveniently carried out in closed vessels in air.

#### Temperature effect

The catalysis proceeded faster at higher temperature, with a concomitant decrease of enantioselectivity (Table 3, entries 12–15). At 60 °C, catalyst decomposition became notable by incomplete conversions (Table 3, entry 15). At temperatures below 0 °C, the catalytic reaction was slow not only because of the thermal effect, but because of the limited solubility of F–TEDA. Additional temperature variation experiments were carried out with the more selective catalyst **K2** and phenyl ester **6** as substrate (Table 3). Results for either fluorination (→**6**-**F**) with F–TEDA or the more soluble reagent NFSI (*N*-fluorobenzenesulfonimide) and NCS (*N*-chlorosuccinimide) for chlorination [43] (→**6**-**Cl**) are displayed in Table 3.

**Table 3:** Effect of the temperature on the halogenation of **6** with catalyst **K2**.^a^

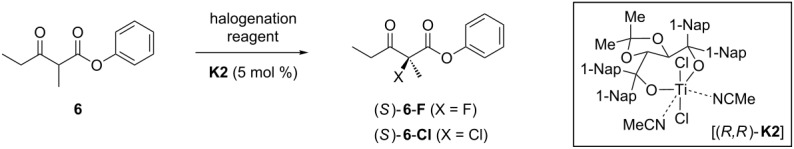						

Entry	Reagent	Solvent	*T* (°C)	*t* (h)	Product	ee (%)

1	F–TEDA	MeCN	0	0.3	**6**-**F**	86
2	F–TEDA	MeCN	rt	0.15	**6**-**F**	88
3	F–TEDA	MeCN	50	<0.3	**6**-**F**	79
4	NFSI	CH_2_Cl_2_	−25	–^b^	**6**-**F**	89^b^
5	NFSI	CH_2_Cl_2_	0	19	**6**-**F**	91
6	NFSI	CH_2_Cl_2_	rt	10	**6**-**F**	87.5
7	NCS	MeCN	rt	0.5	**6**-**Cl**	82
8	NCS	CH_2_Cl_2_	0	7	**6**-**Cl**	90

^a^Reactions were run to complete conversion (TLC), unless otherwise mentioned. ^b^Analysis at 15% conversion. Complete conversion occurred after warming to rt.

Above ambient temperature, increasing temperatures give lower selectivity (Table 3, entries 1–3). Cooling to 0 °C in some cases increased enantioselectivity at the cost of longer reaction times (Table 3, entries 1–3, 4–6, compare also Table 2, entry 12). The optimum temperature depends on substrate, halogenation agent and solvent, but satisfactory results were often achieved at ambient temperature.

#### Solvent effects

The choice of solvent for the catalytic fluorinations with F–TEDA is limited by the solubility of this reagent. Solvents must dissolve the ionic reagent, but not deactivate the Lewis acidic catalyst. Suspensions of F–TEDA in MeCN gave results inferior to saturated F–TEDA solutions in MeCN (*c* = 0.15 mol·L^−1^). Nitromethane dissolved less of the reagent, and the reaction was slower. Aside from water, one of the best solvents for F–TEDA was *N*,*N*-dimethylformamide, however, it deactivated the titanium catalyst. It was possible to study a wider range of solvents in the asymmetric catalytic fluorination by choosing NFSI (*N*-fluorobenzenesulfonimide) as the reagent with better solubility. Reaction with NFSI in dichloroethane gave **1**-**F** with 24% ee (5 mol % **K1**, 40 °C, 3 d, 66% conversion), which is close to the enantiomeric excess observed with F–TEDA in acetonitrile at the same temperature (Table 2, entry 14). The fluorination of **6** with NFSI in several solvents led to similar results in all solvents that supported the reaction (Table 4). In conclusion, MeCN is the preferred solvent for catalytic fluorinations with F–TEDA, whereas CH_2_Cl_2_ is the preferred solvent for fluorinations with NFSI [46].

**Table 4:** Solvent effects on selectivity of fluorination of **6** with NFSI and **K2**.

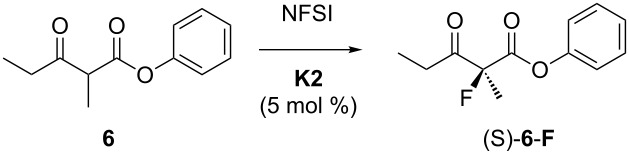			

Entry	Solvent	*t* (h)	ee (%)

1	MeCN	40	84.5
2	CH_2_Cl_2_	10	87.5
3	THF	18^a^	77.5
4	toluene	24	85.6

^a^28% conversion.

#### Variation of the electrophilic fluorinating agent

**Figure 5:**
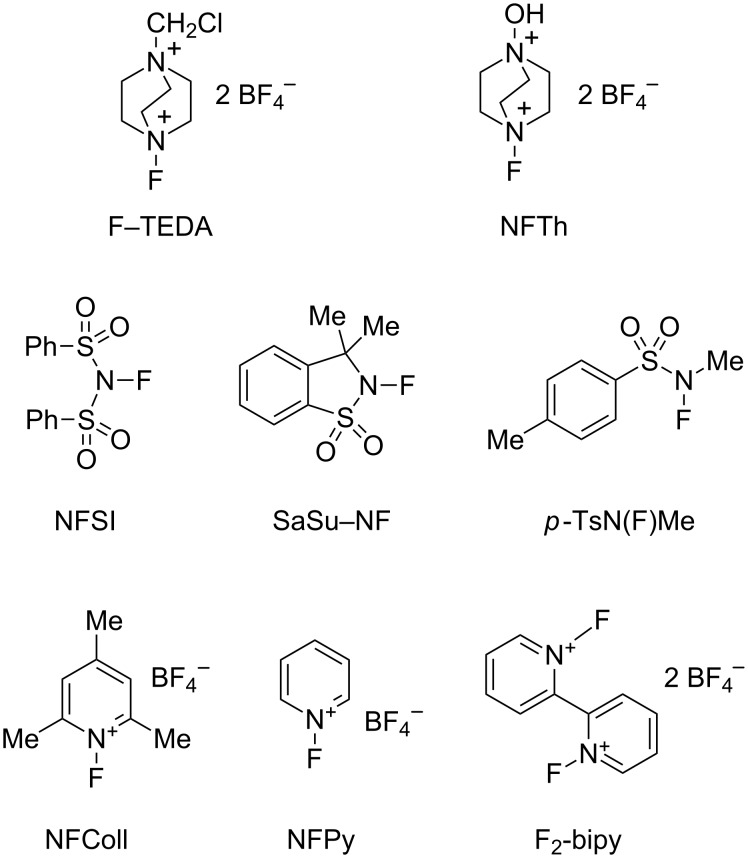
Electrophilic fluorinating reagents of the N–F-type. F–TEDA [27]; NFTh = 1-fluoro-4-hydroxy-1,4-diazoniabicyclo[2.2.2]octane bis(tetrafluoroborate) [84]; NFSI = *N*-fluorobenzosulfonimide [85]; SaSu–NF = saccharine-sultam derived NF reagent (3,3-dimethyl-2,3-dihydro-1,2-benzothiazole-1,1-dioxide) [86–87]; *p*-TsN(F)Me [88]; NFColl = *N*-fluorocollidinium tetrafluoroborate and NFPy = *N*-fluoropyridinium tetrafluoroborate [72,89]; F_2_-bipy = 1,1'-difluoro-bipyridinium bis(tetrafluoroborate) [90].

A selection of commercially available electrophilic fluorinating reagents (Figure 5) was tested in the catalytic reaction in MeCN (Table 5). The dicationic reagents F–TEDA and NFTh [84] (Table 5, entries 1 and 2) were the most reactive. Neutral *N*-fluoro-sulfonamides supported the reaction, but electron-withdrawing groups on nitrogen were needed to induce a fast reaction (Table 5, entries 3, 7 and 8). *N*-fluoropyridinium salts (Table 5, entries 4–6) gave rise to slow catalyses. With the exception of the dicationic *N,N*'-difluorobipyridinium salt (Table 5, entry 6), the reagents induced similar enantioselectivity.

**Table 5:** Effect of the fluorinating agent on the catalytic fluorination of **F1** with 10 mol % **K1**.

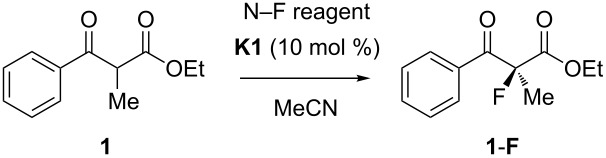				

Entry	Reagent	Time	Conversion (%)	ee (%)

1	F–TEDA^a^	<5 h	>90	28
2	NFTh	<30 h	>90	32.5
3	NFSI	5 d	90	28.0
4	NFPy	5 d	30	28.8
5	NFColl	5 d	<5	–
6	F_2_-bipy	7 weeks	80	≤3
7	*p*-TsN(F)Me	>1 week	0	–
8	SaSu–NF	>1 week	0	–

^a^5 mol % of catalyst.

#### Ligand variations in the asymmetric titanium-catalyzed fluorination reaction

**Scheme 5:**
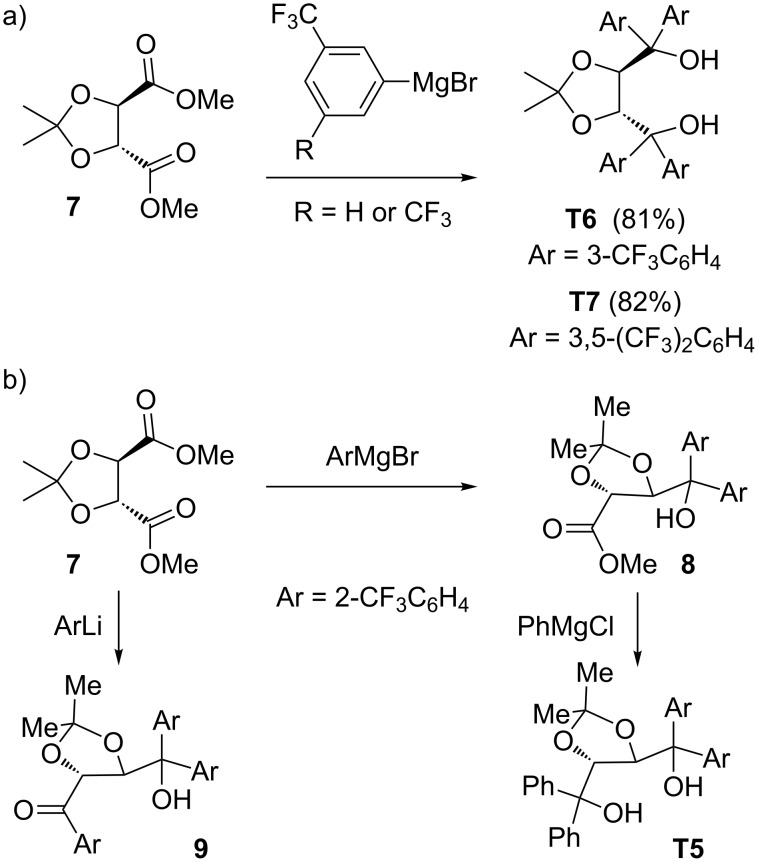
Synthesis of trifluoromethyl-substituted TADDOL ligands.

We tested several chiral diols in the titanium-catalyzed asymmetric fluorination, but notable success was only achieved with TADDOL ligands [91]. We obtained several TADDOLs from commercial sources (**T1**–**T3**) or courtesy of A. K. Beck from the Seebach group (**T4**, **T8**–**T10**). In addition, we prepared trifluoromethylated TADDOLs (Scheme 5), because introduction of fluorine into the ligands was expected to increase the Lewis acidity of the catalyst complex. The syntheses started from dioxolane-diester **7** by reaction with 3-trifluoromethylphenyl or 3,5-bis(trifluormethylphenyl) Grignard reagents in the usual way to give TADDOLs **T6** and **T7** [92] in high yield (Scheme 5a). Interestingly, the reaction of *o*-trifluoromethylphenyl Grignard reagent with **7** gave hydroxyester **8** (Scheme 5b); the latter, with an excess of phenylmagnesium chloride, gave a mixed aryl TADDOL **T5**. For another approach to mixed aryl TADDOLs [93]. The more reactive *o*-trifluoromethylphenyllithium failed to give a TADDOL as well, but instead furnished the hydroxyketone **9** in low yield (Scheme 5b).

The performance of several TADDOLs as ligands in the titanium-catalyzed asymmetric fluorination of the substrates **1**, **10** and **11** to the respective products **1**-**F**, **10**-**F** and **11**-**F** is shown in Table 6. The catalysts of the type [TiCl_2_(TADDOLato)] [94] were all prepared in situ from the ligands and TiCl_2_(OiPr)_2_ in MeCN. To start with, the (*R*)-BINOL-derived complex displayed low activity and selectivity (Table 6, entry 1). The phenyl-TADDOL ligand **T1** induced enantiomeric excesses that increased with the steric requirements of the substrate ester group (Table 6, entry 2). The effect was more pronounced for 1-naphthyl-TADDOL **T2** (Table 6, entry 3), which was the ligand with the best overall performance, displaying both high selectivity and fast kinetics in the catalysis. On the contrary, **T3** with a 2-naphthyl group behaved very much like **T1** towards substrate **1** and was not studied further (entry 4). A further increase in steric requirement and aromatic stacking area was realized with 9-phenanthrenyl-TADDOL **T4**; this gave a maximal enantioselectivity with benzyl ester **10**, rather than with the bulky ester **11**. Presumably, the combined steric effects of substrate and ligand cannot exceed certain optimal limits. In the series of trifluoromethylated TADDOLs **T5**, **T6**, **T7** there was a notable decrease of enantioselectivity and finally an inversion (with substrate **1**) of the sense of induction that roughly correlates with the number of CF_3_ groups in the ligand (Table 6, entries 6–8). Variations of the TADDOLs in the dioxolane backbone had little influence on stereoselectivity (Table 6, entry 2 and 3 versus entry 9 and 10). The presence of an ortho-methoxy group in the TADDOL aryl group was not tolerated (Table 6, entry 11).

**Table 6:** Effects of the variation of the ligand structure and substrate stereochemistry.^a,b^

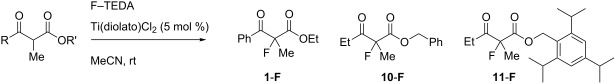					

Entry	Diol ligand		ee (%)		
	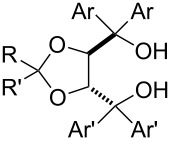		**1**-**F**	**10**-**F**	**11**-**F**

1		(*R*)-BINOL	−1.7^c,d^	n.d.	n.d.
2	**T1**	R,R' = Me; Ar,Ar' = Ph	28.2	30–37	53–55
3	**T2**	R,R' = Me; Ar,Ar' = 1-Nap	57.5	55	85^e^
4	**T3**	R,R' = Me; Ar,Ar' = 2-Nap	26	n. d.	n. d.
5	**T4**	R,R' = Me; Ar,Ar' = 9-Phen	36^f^	74	35^f^
6	**T5**	R,R' = Me; Ar = Ph; Ar' = 2-CF_3_C_6_H_4_	11.5	19	12
7	**T6**	R,R' = Me; Ar,Ar' = 3-CF_3_C_6_H_4_	−5^d^	28	13
8	**T7**	R,R' = Me; Ar,Ar' = 3,5-(CF_3_)_2_C_6_H_3_	−32^d^	11	−12^d^
9	**T8**	R = Me; R' = Ph; Ar,Ar' = Ph	25	32	45
10	**T9**	R = Me; R' = *t-*Bu; Ar,Ar' = 1-Nap	56	56	84
11	**T10**	R,R' = Me; Ar,Ar' = 2-MeOC_6_H_4_	0^f^	0	0

^a^TADDOLs in this table all have (*R*,*R*)-configuration. ^b^Abbreviations: 1-Nap = 1-naphthyl; 2-Nap = 2-naphthyl; 9-Phen = 9-phenanthrenyl; n.d. = not determined. ^c^Reaction for 10 h at 0 °C, conversion not complete. ^d^A negative sign denotes inversion of the excess configuration for the fluorination product (*R*), relative to the excess configuration obtained with **T1** (which is (*S*)). ^e^With pure **K2** instead of the in situ catalyst from **T2**, selectivity is 90% ee. ^f^Conversion stops before completion of the reaction.

#### Assignment of absolute configurations of selected reaction products

**Scheme 6:**
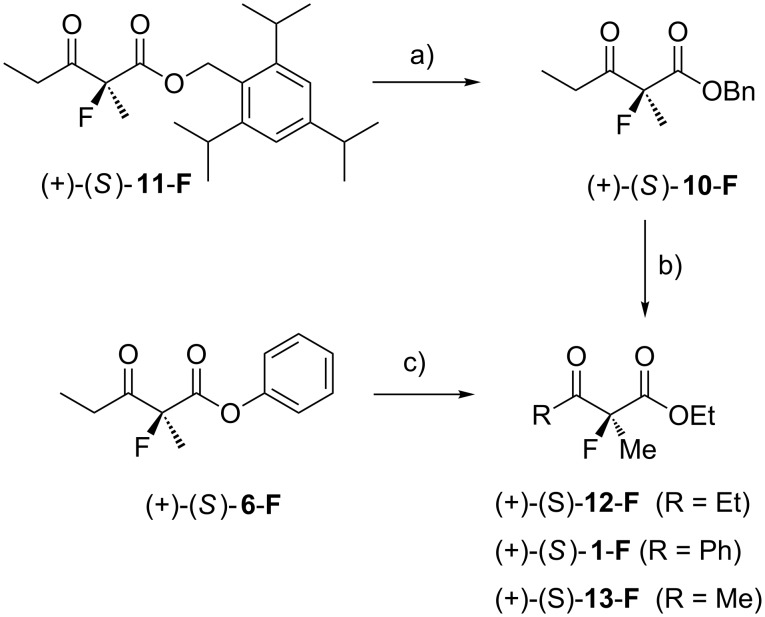
Correlation experiments for the assignment of absolute configuration to fluorination products **11-F**, **10-F** and **6-F**. The absolute configuration of **1-F**, **13-F** and **12-F** in relation to the sign of their optical rotation was established by Kitazume et al. [95] a) 1. BCl_3_, CH_2_Cl_2_, −20 °C, 15 min; 2. cyanuric chloride, PhNMe_2_, BnOH. b) EtOH, cat. Na, rt, 15 min. c) *p*-TsOH, EtOH, rt, 1 d.

The excess configuration of (+)-**1-F** was directly assigned as (2*S*) by comparison with literature data. Kitazume et al. reported [α]_D_ = +85.4 (*c* 1.97, MeOH) for (*S*)-**1-F** with an enantiomeric excess of >98% [95]. Our sample ([α]_D_ = +53.8, (*c* 0.545, MeOH)) had an optical purity of 63%, which is in good agreement with the 62% ee from the HPLC measurement. A sample of the benzyl ester (+)-**10-F** (68% ee by HPLC) was transesterified (EtOH, cat. Na, <5 min at rt) to give the known [95] ethyl ester (+)-(*S*)-**12-F** (67.9% ee by GC). By comparison with the literature optical rotation value, the material had an optical purity of 73%, which is sufficiently close to the enantioselectivity determined by GC, considering the difficulties of purifying this volatile compound on a small scale (Scheme 6). A sample of (+)-**11-F** of 85.6% ee (by HPLC) was treated with BCl_3_ and the resulting carboxylic acid esterified in situ with cyanuric chloride, *N*,*N*-dimethylaniline and benzyl alcohol yielding (+)-**10-F** in 77% yield (84.9% ee by HPLC), whose configuration has already been established by correlation with **12-F**. Comparison of the elution order on a chiral HPLC column with the (+)-sign of the optical rotation implied an (*S*)-configuration of this sample, and therefore likewise for (+)-**11-F**. In addition, a sample of (+)-**6-F** was transesterified in acidic ethanol to **12-F**. Assignment of the retention time of the major enantiomer in GC allowed the assignment of the (*S*)-configuration to (+)-(*S*)-**6-F** (Scheme 6). So far, samples emerging from our catalytic fluorination with a catalyst incorporating (*R*,*R*)-TADDOL ligands have displayed positive optical rotation and a (2*S*)-configuration in the excess enantiomer.

#### Mechanistic considerations

**Scheme 7:**
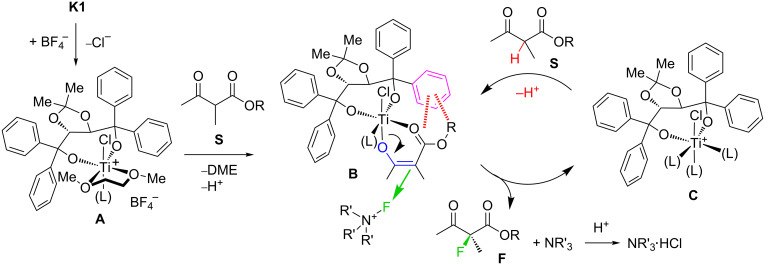
Mechanistic scheme proposed, based on visual and spectroscopic observations. L = solvent, counterions.

The addition of TiCl_4_ to acetyl acetone (2,4-pentanedione) gives rise to the red-colored chelate complex TiCl_2_(acac)_2_ (acac = acetylacetonate, κO, κO'-2,4-pentanedionate) [96–97]. The complex [TiCl(κO,κO'-1,3-diphenyl-1,3-propanedionate)(TADDOLate)(THF)], derived from dibenzoylmethane, was described as being yellow [98]. When the complex **K1** was mixed with β-ketoester substrates (**S**) in acetonitrile solution, yellow- or red-colored solutions were formed within few minutes, which implies that a β-diketonate complex was formed. We therefore assume that the catalytic acceleration of the electrophilic fluorination reaction with F–TEDA by Lewis acidic titanium complexes is due to the intermediacy of chelating titanium–enolate complexes **B** (Scheme 7).

**Figure 6:**
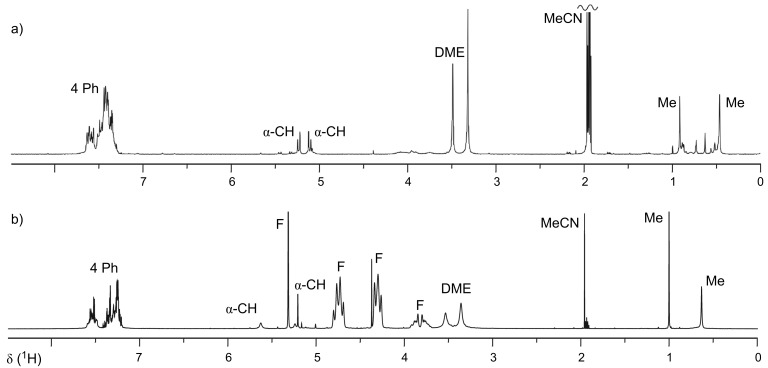
^1^H NMR spectra of a species of the type **A**, generated in CD_3_CN solution from **K1** by ionization in the presence of: a) AgBF_4_, or: b) excess F–TEDA reagent. Assignments: α-CH = dioxolane H-C(4) and H-C(5). F = signals due to F–TEDA (and some H–TEDA). DME = coordinated 1,2-dimethoxyethane. Me = dioxolane methyl-C(2) groups.

The intermediacy of cationic species **A** is postulated based on NMR experiments: A solution of **K1** in CDCl_3_ did not show any signs of ionization and kept the spectral properties expected for a *C*_2_ symmetric species down to 230 K. The limited solubility of **K1** in CD_3_CN prevented low temperature NMR experiments in that solvent. However, the reaction of **K1** with an equivalent of AgBF_4_ in CD_3_CN produced the signals of species **A** (Scheme 7) corresponding to the formula [TiCl(TADDOLato)(DME)(MeCN)]BF_4_, which is characterized by two signals for diastereotopic geminal methyl groups (δ = 0.48 and 0.93 ppm), and two doublets for the diastereotopic methyne hydrogens (δ = 5.08 and 5.20 ppm, *J* = 7.2 Hz) of the TADDOLate backbone (Figure 6a). This monocationic species could not be isolated; after evaporation and redissolution in CDCl_3_, the sample had lost DME (1,2-dimethoxyethane) and displayed signals corresponding to the free TADDOL ligand, implying decomposition of the complex. Still we were able to show that a silver cation is not needed for ionizing the catalyst under these reaction conditions: When a suspension of **K1** was stirred with excess solid F–TEDA in CD_3_CN, and the suspension filtered, the ^1^H NMR spectrum of the filtrate displayed signals for a similar cationic species corresponding to **A**, in addition to signals from the F–TEDA cation (Figure 6b). The spectrum of this *C*_1_-symmetric species displayed signals for the diastereotopic methyl groups (δ = 0.64 and 1.01 ppm), and two broadened signals for the diastereotopic methine hydrogens (δ = 5.25 and 5.65 ppm) of the TADDOLate backbone. The signals for ligated DME showed considerable broadening. The chemical shift difference observed may either be explained by a fast equilibrium between [TiCl(TADDOLato)(DME)(MeCN)]BF_4_ and [TiCl(BF_4_)(TADDOLato)(DME)], or through the effects of ion pairing and variable concentrations.

**Figure 7:**
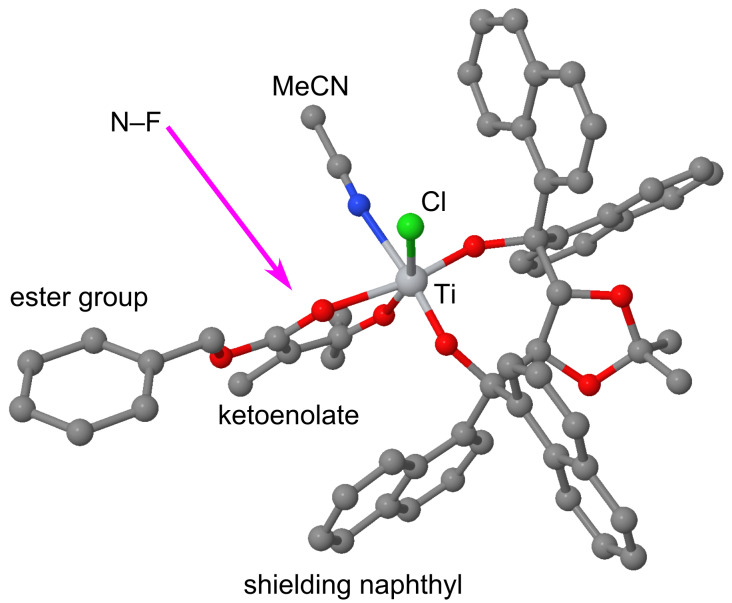
Steric model explaining the face selectivity observed in the titanium–TADDOLate complex catalyzed fluorination. The structural model of this complex [TiCl(TADDOLate)(β-ketoenolate)(MeCN)] of the type **B** (Scheme 7) was obtained by combining X-ray data of **K2** with a modeled structure of the enol of substrate **10**. The arrow symbolizes the external attack of the fluorinating reagent N–F.

These experiments show that ionization of **K1** to generate **A**, under the conditions of the catalytic reaction, readily takes place. In a second step, we assume that **A** undergoes ligand-exchange reactions with coordination of a substrate **S** for DME, followed by loss of the α-carbonyl hydrogen as H^+^ to give a mono-ketonate complex **B**. The proton may combine with chloride counterion to give HCl, which is only weakly dissociated in acetonitrile (the p*K*_a_ of HCl in MeCN has recently been measured as 10.6) [99]. Complex **B** would then be attacked by external F–TEDA reagent at the central carbon of the enolate, diastereoselectively, from the face opposite to a shielding TADDOLate aryl group (Scheme 7, **B**→**C**). In order to arrive at a simple structural model of **B**, we used the coordinates from the X-ray crystal structure of the 1-naphthyl-TADDOL complex **K2** [83] for the [TiCl(TADDOLate)(MeCN)] fragment and coordinates for the enol of benzyl ester substrate **10**, generated with the simple model-building software ALCOGEN [100], for the ketoenolate fragment. The two substructures were joined manually, such that the enolate oxygen occupies the axial position trans to chlorine at titanium, and the carbonyl oxygen occupies the position of a dissociated acetonitrile ligand. The resulting model is shown in Figure 7. The model nicely illustrates the shielding function of a face-on 1-naphthyl group, which may direct the attack of the incoming fluorinating reagent.

**Figure 8:**
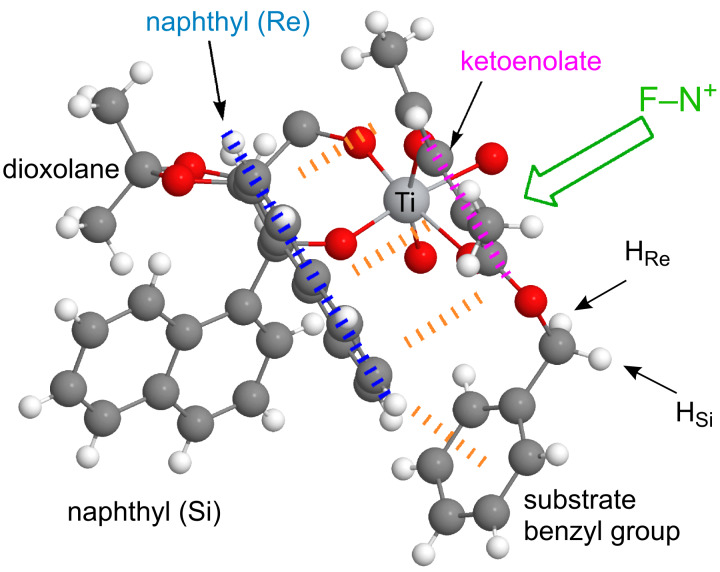
Excerpt from the X-ray structure of a catalyst/substrate complex [Ti(1-naphthyl-TADDOLato)(β-ketoenolate)_2_] [102]. Two important attractive interactions between substrate and TADDOL ligand fragments can be identified: 1. Face-to-face arrangement of the ligand 1-naphthyl group and the substrate ketoenolate plane; 2. edge-to-face stacking interaction between the ligand naphthyl and the substrate benzyl ester groups.

This hypothesis was used as a starting point for more detailed studies; QM/MM calculations on the postulated intermediary isomeric β-ketoenolate complexes and their attack by the N–F reagent were carried out, as described elsewhere [101]. Among the eight diastereomeric forms of **B**, the most stable complex is indeed the one predicted by the simple steric model (Figure 7), which is expected to produce the fluorination product **F** with (*S*)-configuration, as experimentally observed [101]. More recent experimental studies led to the isolation of complexes [Ti(1-naphthyl-TADDOLato)(β-ketoenolate)_2_], which were characterized by X-ray crystallography in the solid state and by NMR spectroscopy in solution [102]. In either mono- or bis(ketonato) complex, the shielding of one diastereomorphic face of the coordinated enolate by a 1-naphthyl group of the ligand was found. Either a *C*_1_-symmetric mono-ketonato or a *C*_2_-symmetric bis(ketonato) complex will react with F–TEDA to give the fluorinated β-ketoester product with the same sense of induction as experimentally found in the catalytic reaction [102]. In the X-ray structure of the bis(enolate) of benzyl 2-methyl-3-oxopentanoate [102] (Figure 8), the face-to-face alignment of the substrate enolate, which directs the attack of the incoming electrophile to the other face, is clearly an important element. However, closer inspection also reveals an attractive edge-to-face CH-to-arene-π interaction between the substrate benzyl ester and the ligand naphthyl group, which was not predicted by the simplistic steric models in Figure 6. This attractive interaction may be responsible for the generally higher level of enantioselectivity observed in the fluorination of benzyl and aryl esters [57] when compared to substrates containing aliphatic alkyl ester groups [40].

A subsequent paper will concentrate on the substrate scope of the titanium–TADDOLate-catalyzed asymmetric fluorination of β-ketoesters and its extension to other activated carbonyl compounds as substrates [57].

## Supporting Information

File 1 Experimental procedures and characterization data.
