# Supplementary material for: Development of the titanium–TADDOLate-catalyzed asymmetric fluorination of β-ketoesters
Source: Beilstein J Org Chem. 2011 Oct 17;7:1421–35. doi: 10.3762/bjoc.7.166 (PMC3201056; doi:10.3762/bjoc.7.166)
Supplement: File 1 — Experimental procedures and characterization data. [file Beilstein_J_Org_Chem-07-1421-s001.pdf]

Supporting Information

for

Development of the titanium–TADDOLate-catalyzed asymmetric fluorination of β-ketoesters

Lukas Hintermann[1,2], Mauro Perseghini[1] and Antonio Togni*[1]

Address: [1]Laboratorium für Anorganische Chemie, ETH Zürich, Wolfgang-Pauli-Str. 10, 8093 Zürich, Switzerland and [2]Department Chemie, Technische Universität München, Lichtenbergstr. 4, 85748 Garching bei München, Germany

Email: Antonio Togni - togni@inorg.chem.ethz.ch

* Corresponding author

Experimental procedures and characterization data

Contents

**1. General**	S 2
**2. Catalytic fluorination of β-ketoesters**	S 2
**3. Reactivity and substrate screening**	S 3
**4. Preparative catalytic fluorination**	S 4
**5. Synthesis of TADDOL ligands**	S 6
**6. Ligand-screening experiments**	S 12
**7. Stereochemical-correlation experiments**	S 13
**8. Mechanistic experiments**	S 16

1. General

Reactions were carried out under an argon atmosphere. F-TEDA and NCS were obtained from Aldrich. Column chromatography was performed with silicagel 60 (Fluka, particle size 40–63 μm). NMR spectra were recorded in $CDCl_3$ at ambient temperature (20–25 °C) relative to TMS, unless otherwise noted. ^{13}C NMR shifts are relative to TMS, but referenced through solvent peaks. ^{19}F NMR spectra are referenced against internal $CFCl_3$. MS spectra and elemental analyses were carried out by in-house services at ETH Zürich. Enantiomer excesses were determined by HPLC or GC analyses on DAICEL CHIRALCEL columns with hexane/2-propanol as mobile phase. GC analyses were performed on SUPELCO β-DEX 120 (30 m) or γ-DEX 120 (30 m) columns, helium carrier (1.4 mL/min), split injector (42 mL/min, 200 °C), FID detector (Air/H_2 350/35 mL/min, 250 °C). Data for individual compounds are found in a subsequent paper [1]. **Abbreviations**: "sat." = saturated solution of, aq = dissolved in water, *t*-BuOMe is *tert*-butyl-methylether. F-TEDA is 1-chloromethyl-4-fluoro-1,4-diazoniabicyclo[2.2.2]octane bis(tetrafluoroborate); the BF_4-salt was used throughout this work. THF is tetrahydrofuran. CC is column chromatography (on SiO_2).

2. Catalytic fluorination of β-ketoesters

Preparation of a saturated solution of F-TEDA

In a 100 mL brown glass bottle, acetonitrile (analytical reagent grade) was added to finely powdered F-TEDA (1-chloromethyl-4-fluoro-1,4-diazoniabicyclo[2.2.2]octane bis(tetrafluoroborate); "Selectfluor"; 6.0 g) and granular molecular sieves 3 Å (10 g) up to the 100 mL mark. The contents were stirred magnetically for 1 h. The bottle was set aside for sedimentation of the suspended particles. The supernatant, a saturated solution of the F-TEDA, had an approximate concentration of 0.145 mol/L (according to iodometric titration) [2] and was used for catalytic reactions. Notes: The solution had a shelf life of at least 2 weeks. Occasionally, the solution became turbid (opalescent) and gave unsatisfactory performances in catalysis.

General procedure for catalytic fluorinations (GP1)

To the substrate (0.25 mmol) in MeCN (1 mL), the catalyst was added (0.0125 mmol, 5 mol %) and the mixture stirred until a clear solution had formed. Optionally, molecular sieves or other additives are added at this point. The reaction was started by addition of a saturated F-TEDA solution (0.145

M in MeCN; 2.0 mL, 0.29 mmol; ≈ 1.15 equiv). The reaction mixture was stirred at r.t. until complete conversion (TLC check), or until no further change was observed.

Workup: The reaction mixture was flushed into a separatory funnel with *t*-BuOMe (30–50 mL) and water (50–100 mL). The aqueous phase was discarded and the organic phase filtered over a small plug of neutral/deactivated Al_2O_3 on cotton. The filtrate was evaporated and the residue analyzed by chiral HPLC/GC. Yields and additional analytical data were obtained after further purification by CC.

General procedure for catalytic halogenations (fluorination, chlorination) with nonionic reagents: (GP2)

Given the good solubility of the respective reagents, catalytic fluorinations with *N*-benzenesulfonimide (NFSI) or chlorinations with *N*-chlorosuccinimide (NCS) were readily carried out in a range of solvents: A mixture of the substrate (0.25 mmol) in the solvent (1 mL) was stirred with the catalyst until the substrate was dissolved. The solid halogenating reagent (0.275–0.325 mmol; 1.1–1.3 equiv) was added and the reaction stirred at r.t.; see GP1 for workup and purification.

3. Reactivity and substrate screening

Experiments were performed according to the GP1. For analysis, samples of ca. 0.1 mL were removed and diluted with $CDCl_3$ (1.0 mL). The mixture was filtered (cotton plug/Pasteur pipette) directly into an NMR tube for analysis (^{1}H, ^{19}F NMR). The catalysts **K1** and **K2** were prepared according to the published procedures [3]. For the synthesis and characterization of the substrates **1**, **2**, **4**, **6**, **10** and **11** see the subsequent paper [1]. Details of the characterization of the fluorination products **1-F**, **2-F**, **4-F**, **6-F**, **10-F**, **11-F, 12** and the chlorinated product **6-Cl** are also included in the subsequent paper [1]. Ethyl phenylcyanoacetate (**3**) was synthesized according to a literature procedure [4]. The fluorination product **3-F** is a known compound [5]. Dimethyl (*R*,*R*)-2,3-*O*-isopropylidene-tartrate (**7**) was available from Fluka. **Chiral analysis:** For conditions of the analysis by chiral HPLC or chiral GC, see the subsequent paper [1].

4. Preparative catalytic fluorination

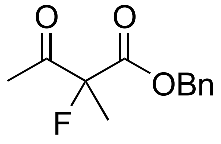

Benzyl 2-fluoro-2-methyl-3-oxobutanoate (4-F). To a stirred mixture of **4** (206 mg, 1.00 mmol) and finely powdered F-TEDA (380 mg, 1.07 mmol) in MeCN (5 mL) at r.t., a solution of $TiCl_4$ (0.13 M in MeCN, 0.50 mL, 0.065 mmol, 6.5 mol %) was added dropwise, generating a red-brown coloration. After being stirred for 4 h at r.t. the then virtually colorless suspension was taken up in water/*t*-BuOMe. The organic phase was washed with water (3 x) and dried over $MgSO_4$. Filtration and evaporation gave 228 mg of a yellowish oil, which was filtered through neutral, deactivated Al_2O_3 with *t*-BuOMe/pentane (1:1, 5 mL). Evaporation gave the pure product as a colorless oil (198 mg, 96%). See subsequent paper for characterization data [1].

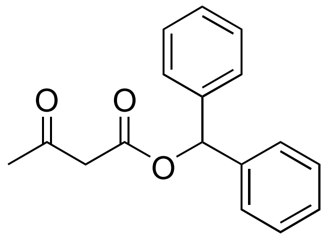

Diphenylmethyl 3-oxobutanoate (5) [6]. To diphenylmethanol (2.745 g, 14.9 mmol) in CH_2Cl_2 (5 mL) at 0 °C, 1.25 mL diketene (1.25 mL, 16.3 mmol) was slowly added. After addition of a single drop of CF_3COOH and some DMAP (15 mg), an exothermic reaction commenced and the solvent started to boil despite cooling with ice (CAUTION; it is advisable to use more solvent and to add the diketene dropwise with stirring after addition of the catalyst to prevent an exothermic run-over). After 5 min, analysis by TLC indicated that all starting material had been consumed (*t*-BuOMe/hexanes 1:2; Ph_2CHOH R_f = 0.53, product R_f = 0.46). After extraction with CH_2Cl_2/water, the organic phase was washed with sat. $NaHCO_3$ and dried over Na_2SO_4. Evaporation gave 4.085 g of a yellow oil. Purification by CC (*t*-BuOMe/hexanes 1:10–1:5) gave 3.92 g (98%) of a colorless oil, which crystallized after standing in the refrigerator.

R_f = 0.46 (*t*-BuOMe/hexanes 1:2); m.p.: 38.5–39.8 ˚C; ^{1}H NMR (300 MHz): δ = 2.16 (s, 3 H, MeCO), 3.50 (s, 2 H, CH_2), 6.92 (s, 1 H, $OCHPh_2$), 7.20–7.37 (m, 10 H_{Ar}) ppm; (enol, 10%): δ = 1.92 (s, 3 H, MeCO), 5.14 (br. s, 1 H, H-C=), 6.91 (s, 1 H, $OCHPh_2$), 7.20–7.37 (m, 10 H_{Ar}), 11.97 (s, 1 H, OH) ppm; ^{13}C NMR (75.5 MHz): δ = 29.9, 50.1, 77.8, 127.0, 128.0, 128.4, 139.4, 166.0, 200.0 ppm; (enol): δ = 21.1, 76.3, 89.6, 126.9, 127.8, 128.4, 140.0, 171.5, 176.1 ppm ; IR (film, NaCl): ν = 3063w, 3031w, 2930w, 1745s, 1718s, 1495w, 1455w, 1407w, 1360m, 1317m, 1258m, 1147m, 1028w, 971w, 750m, 699m cm^{-1}; MS (EI): *m*/*z* (%) = 268 (2) M^+, 250 (5), 224 (8), 208 (3), 183 (66), 167 (100), 152 (19); analysis calcd (%) for $C_{17}H_{16}O_3$ (268.31): C 76.10, H 6.01; found: C 75.92, H 6.14.

Diphenylmethyl 2-fluoro-3-oxobutanoate (5-F) and **Diphenylmethyl 2,2-difluoro-3-oxobutanoate (5-FF)**:

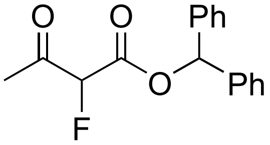

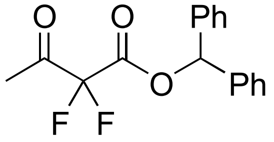

a) According to GP1 from **5** (0.25 mmol) with $CpTiCl_3$ (5.3 mol %) as catalyst and 1.8 mL of sat. F-TEDA in MeCN (0.261 mmol). Reaction time: 5.5 h. Crude: 73 mg of **5-F/5-FF** (11:1 by ^{1}H NMR) at 90% conversion. b) According to GP1 form **5** with **K1** (5.4 mol %), 1.8 mL sat. F-TEDA in MeCN (0.261 mmol). Reaction time: 5.5 h. Crude: 71 mg of **5-F/5-FF** (8:1 by ^{1}H NMR) at 87% conversion. c) In analogy to GP1 from **5** (298 mg, 1.11 mmol) with **K2a** (5 mol %), 200 mg of powdered molecular sieves (3 Å), 7.8 mL sat. F-TEDA in MeCN (1.13 mmol). Reaction time: 2.5 h. Purification by CC (*t*-BuOMe/hexanes = 1:10) gave 231 mg (ca 72%) of **5-F**/**5-FF** (6:1).

R_f = 0.48 (*t*-BuOMe/hexanes 1:10); ^{1}H NMR (300 MHz), **5-F** (82.8%): δ = 2.28 (d, J(F,H) = 4.2 Hz, 3 H, MeCO), 5.28 (d, J(F,H) = 49.6 Hz, 1 H, CHF), 6.98 (s, $CHPh_2$), 7.27–7.42 (m, 10 H_{Ar}) ppm; (enol, 3.4%): δ = 2.06 (dd, J = 4.4, 0.7 Hz, 3 H, MeC–OH), 10.00 (d, J(F,H) = 4.1 Hz, 1 H, OH) ppm; **5-FF** (13.8%): δ = 2.37 (t, J(F,H) = 1.7 Hz, 3 H, MeCO), 6.99 (s, 1 H, $CHPh_2$), 7.27–7.42 (m, H_{Ar}) ppm; ^{13}C NMR (50.3 MHz), **5-F**: δ = 26.0, 79.2 (d, J(F,C) = 1 Hz), 91.5 (d, J(F,C) = 199 Hz), 126.8–128.7 (several CH), 138.8, 138.8, 163.1 (d, J(F,C) = 24 Hz), 198.7 (d, J(F,C) = 24 Hz) ppm; ^{19}F NMR (188.3 MHz), **5-F**: δ = –193.2 (dq, J(F,H) = 49.5, 4.2 Hz) ppm; **5-FF**: δ = –114.0 (q, J(F,H) = 1.6 Hz) ppm; MS (HR-MALDI): calcd for [**5-F**+Na]$^+$ 309.090; found 309.089.

Phenyl 2-chloro-2-methyl-3-oxo-pentanoate (**6-Cl**). Prepared according to the GP2. See the subsequent paper for full data [1].

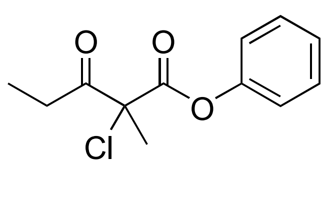

Phenyl 2-fluoro-2-methyl-3-oxo-pentanoate (**6-F**). Prepared according to the GP1. See the subsequent paper for full data [1].

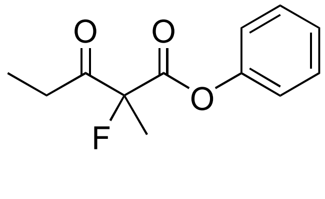

5. Synthesis of TADDOL ligands

Dimethyl (*R*,*R*)-2,3-di-*O*-isopropylidene-tartrate (**7**) was prepared according to a literature procedure [7] or obtained commercially. For the synthesis of TADDOLs, general procedures from the Seebach group were followed [7,8].

T1: α,α,α',α'-Tetraphenyl-(2,2-dimethyl-1,3-dioxolane-4,5-diyl)-dimethanol: Obtained commercially, or according to [7].

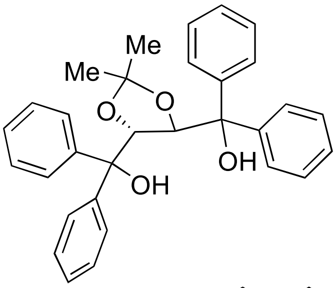

T2: α,α,α',α'-Tetra-1-naphthyl-(2,2-dimethyl-1,3-dioxolane-4,5-diyl)-dimethanol: From Aldrich.

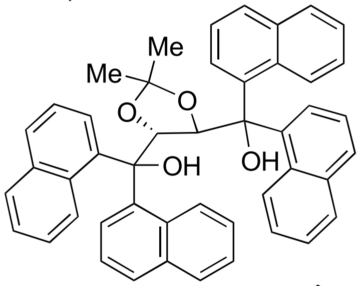

T3: α,α,α',α'-Tetra-2-naphthyl-(2,2-dimethyl-1,3-dioxolane-4,5-diyl)-dimethanol: From Fluka.

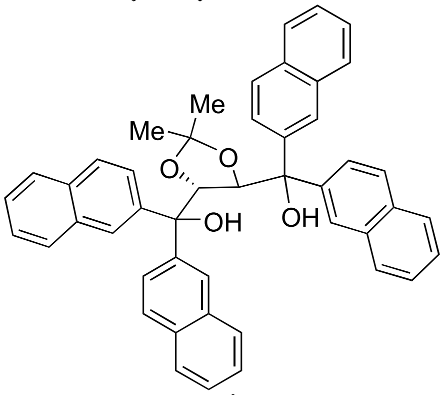

T4: α,α,α',α'-Tetra-9-phenanthrenyl-(2,2-dimethyl-1,3-dioxolane-4,5-diyl)-dimethanol: Obtained by courtesy of Albert K. Beck (Seebach group, ETH Zürich).

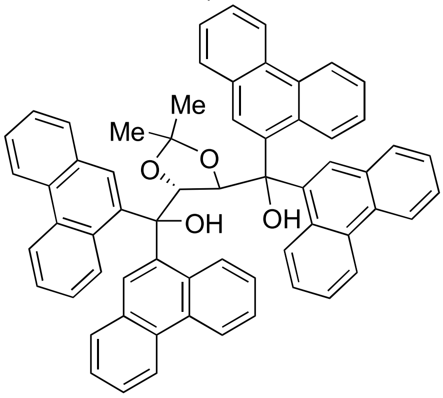

T5 was synthesized from **7** via **8**: **Methyl (4*R*,5*R*)-5-[hydroxybis(2-(trifluoromethyl)phenyl)methyl]-2,2-dimethyl-1,3-dioxolane-4-carboxylate (8)**. A Grignard solution was prepared from 2-bromobenzotrifluoride (9.5 mL, 70 mmol) and magnesium turnings (1.8 g, 74 mmol), in THF (50 mL) at ca. 10 °C. After addition of the aryl bromide, the reaction mixture was stirred for 3 h at r.t. To the dark-brown solution, dimethyl (*R*,*R*)-2,3-*O*-isopropylidene tartrate (**7**) (3.275 g, 15.0 mmol) was added dropwise at 0 °C over 20 min. Within 2 h the reaction mixture was warmed to r.t. and stirred overnight. Careful quenching with sat. NH_4Cl aq (40 mL), HCl aq (5 mL, 2 M) and *t*-BuOMe (150 mL) was followed by washing of the org. phase with KOH (2 x 30 mL, 2 M) and sat. NaCl aq. After drying ($MgSO_4$) and evaporation, the residue was purified by CC (*t*-BuOMe/hexane 1:8). The combined product fractions crystallized on evaporation of the solvents to ca. 5 mL. The yellow crystals were washed with pentane and dried in HV (2 h, 60 °C) to give 3.716 g (52%).

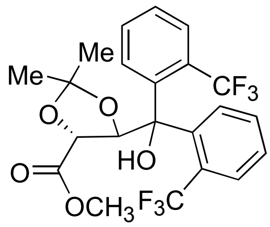

R_f = 0.27 (*t*-BuOMe/hexane 1:5); m.p. 114.7–116.6 °C; $[\alpha]_D$ = +3.2 (c = 1.39, MeOH); ^{1}H NMR (200 MHz): δ = 1.51 (q, *J*(H,H) = 0.6 Hz, 3 H), 1.57 (q, *J*(H,H) = 0.6 Hz, 3 H), 3.29 (s, 3 H), 3.68 (s, 1 OH), 4.45 (d, *J*(H,H) = 7.3 Hz, 1 H), 5.55 (d, *J*(H,H) = 7.3 Hz, 1 H), 7.35–7.49 (m, 2 H), 7.51–7.75 (m, 4 H), 7.88–7.97 (m, 1 H), 8.34 (br. s, $\Delta_{50\%}$ = 34 Hz, 1 H) ppm; ^{13}C NMR (50.3 MHz): δ = 26.2, 26.7, 52.1, 75.4, 81.6 (br.), 112.2, 123.5 (q, *J*(F,C) = 274 Hz), 123.9 (q, *J*(F,C) = 275 Hz), 127.1 (br q, *J*(F,C) ≈ 5 Hz), 128.1, 128.4, 128.6 (q, *J*(F,C) = 7 Hz), 129.7 (q, *J*(F,C) = 2 Hz), 129.7 (q, *J*(F,C) = 32 Hz), 130.4, 130.9 (br.), 131.0 (q, *J*(F,C) = 35 Hz), 131.5, 138.9 (br.), 140.1 (br.), 169.9 ppm; ^{19}F NMR (188.3 MHz): δ = –55.3 (br. s), –55.6 (s) ppm; IR (KBr): ν = 3527m, 2995w, 2962w, 1752s, 1604w, 1493w, 1448m, 1386m, 1377m, 1341m, 1310s, 1162s, 1130s, 1113s, 1063s, 1040s, 1031s, 772m, 768m, 681m, 665m cm^{-1}; MS (HR-MALDI): *m*/*z* = 501.112 (calcd for $[M+Na]^+$: 501.111); analysis calcd (%) for $C_{22}H_{20}F_6O_5$ (478.392): C 55.24, H 4.21; found C 55.29, H 4.42.

((4*R*,5*R*)-5-(Hydroxybis(2-(trifluoromethyl)phenyl)methyl)-2,2-dimethyl-1,3-dioxolan-4-yl)diphenylmethanol (T5). To 35 mL PhMgBr (Fluka, 1 M in THF, 35 mmol), solid ester **8** (3.608 g, 7.54 mmol) was added in portions. The reaction mixture was cooled in a water bath (r.t.). After being stirred for 20 min, TLC analysis indicated complete conversion. The reaction mixture was diluted with *t*-BuOMe (150 mL) and quenched by addition of sat. NH_4Cl *aq* (50 mL). The organic phase was washed with sat. NH_4Cl *aq* (2 x), and water (1 x). After drying (Na_2SO_4) and evaporation, the residue was purified by CC (*t*-BuOMe/hexane 1:15–1:10). The product fractions were taken up in 3 mL of toluene, evaporated

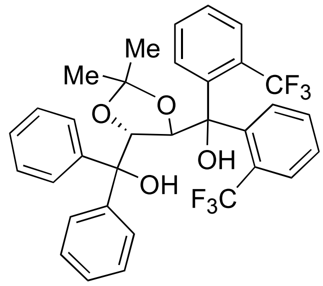

and dried in HV (60 °C, 1 d) to give yellow foam of 3.920 g ($C_{33}H_{28}F_6O_4$+0.3 $PhCH_3$, M_r = 630.205; 82%), still containing 0.3 equivalents of toluene (^{1}H NMR, EA).

R_f = 0.38 (*t*-BuOMe/hexane 1:5); $[\alpha]_D$ = +30.2 (c = 1.81, MeOH; mass includes toluene); ^{1}H NMR (300 MHz, 60 °C): δ = 0.72 (s, 3 H), 1.29 (br. s, 3 H), 4.7 (br. s, $\Delta_{50\%}$ = 180 Hz, 2 OH), 5.04 (br. s, $\Delta_{50\%}$ = 60 Hz, 1 H), 5.19 (d, *J*(H,H) = 7.2 Hz, 1 H), 7.08–7.16 (m, 1 H), 7.16–7.29 (m, 7 H), 7.31–7.43 (m, 6 H), 7.44–7.53 (m, 1 H), 7.60–7.76 (m, 2 H), 7.97–8.11 (m, 1 H) ppm, additional peaks due to toluene (0.3 eq.); ^{13}C NMR (75.5 MHz, 60 °C): δ = 26.9, 27.9, 78.9, 79.4, 81.0, 82.6 (br.), 110.5 (br.), 124.2 (q, *J*(F,C) = 274 Hz), 127.2, 127.4, 127.7, 127.8, 128.0 (q, *J*(F,C) = 7 Hz), 128.3, 128.8 (q, *J*(F,C) = 7 Hz), 129.1, 129.7 (br.), 130.4, 130.7 (br.), 131.5 (br.), 142.3, 146.5 ppm ; additional peaks due to toluene: δ = 21.3, 125.3, 128.3, 129.1; as a result of line broadening, which was still present at 60 °C, not all signals could be detected; ^{19}F NMR (188.3 MHz, r.t.): δ = –53.8 (br. s), –55.0 (br. s) ppm; IR (KBr): ν = 3552w (br.), 3375m (br.), 3061w, 3028w, 2990w, 2936w, 1955w, 1603w, 1495w, 1447m, 1393w, 1373w, 1307s, 1141s, 1065m, 1036s, 882m, 765m, 735m, 702m cm^{-1}; MS (ESI): neg. mode: *m/z* = 601.4 $[M–H]^-$; pos. mode: *m/z* = 620.4 $[M+NH_4]^+$; analysis calcd (%) for $C_{33}H_{28}F_6O_4$+0.3 $PhCH_3$ (630.205): C 66.89, H 4.86; found C 66.89, H 5.02.

VT-NMR revealed the presence of two conformers in the ratio 67:33 (273 K), 71:29 (253 K), 76:24 (233 K), 77:23 (223 K): ^{1}H NMR (300 MHz, 233 K), isomer ratio 3.1:1 (76:24); major conformer: δ = 0.40 (br. s, 3 H; Me), 1.48 (br. s, 3 H; Me), 3.06 (br. s, 1 OH), 4.63 (d, *J*(H,H) = 8.0 Hz, 1 CH), 5.18 (d, *J*(H,H) = 7.9 Hz, 1 CH), 6.49 (br. s, $\Delta_{50\%}$ = 52 Hz, 1 OH), 6.92–7.98 (m, 19 H-Ar), 8.49 (br. s, $\Delta_{50\%}$ = 50 Hz, 1 H_{Ar}) ppm; minor conformer: δ = 0.01 (s, 1 OH), 0.61 (s, 3 H), 1.48 (s, 3 H), 3.82 (s, 1 OH), 5.12 (d, *J*(H,H) = 6.5 Hz, 1 H), 5.53 (d, *J*(H,H) = 6.6 Hz, 1 H), 6.92–7.98 (m, 19 H), 8.40 (d, *J*(H,H) = 8.2 Hz, 1 H) ppm; ^{13}C NMR (75.5 MHz, 223 K): δ = 25.4 (br.), 27.1, 27.8 (br.), 28.5, 77.8, 78.1, 79.1, 79.6, 80.5 (br.), 82.3 (br.), 109.0 (br.), 111.8, 124.1 (q, *J*(F,C) = 272 Hz), 124.5 (q, *J*(F,C) = 272 Hz), 126.5–132.6 (several s and m), 137.9, 140.1 (m), 140.7 (br.), 141.9, 143.6 (br.), 144.0 (br.), 146.0 (br.), 146.4 ppm.

On attempted synthesis of the tetrakis-ortho-trifluoromethyl phenyl substituted TADDOL from **7** and ArLi, the ketoalcohol **9**, incorporating only three aryl groups, was obtained:

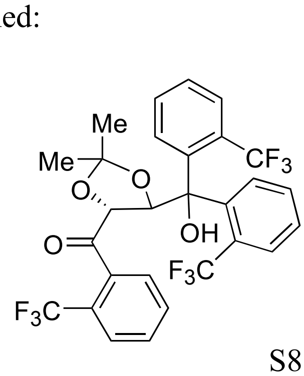

((4*R*,5*R*)-5-(Hydroxybis-(2-(trifluoromethyl)phenyl)methyl)-2,2-dimethyl-1,3-dioxolane-4-yl)-(2-(trifluoromethyl)phenyl)methanone (9). A solution of 2-bromobenzotrifluoride (9.02 g, 40 mmol) in THF (30 mL) was metallated with *n*-BuLi (2 M, pentane; 20 mL, 40 mmol) at –78 °C.

After stirring for 15 min, dimethyl (*R*,*R*)-isopropylidene-tartrate (**7**) (1.65 mL, 9.0 mmol) was added dropwise and the reaction mixture was warmed from –78 °C to r.t. overnight. Workup (sat. NH_4Cl aq, *t*-BuOMe) followed by washing of the organic phase (sat. NaCl aq, 0.2 M HCl aq, sat. NaCl aq) gave a residue after evaporation, which was separated by CC (*t*-BuOMe/hexane 1:10) to give, as the only identified main product, a slowly crystallizing yellow solid (959 mg, 18%); at least 5 additional product spots were detected by TLC.

R_f = 0.25 (*t*-BuOMe/hexane 1:5); ^{1}H NMR (200 MHz, d_6-DMSO, 90 °C, solvent peak = 2.50 ppm): δ = 1.32 (s, 3 H), 1.42 (s, 3 H), 5.25 (d, *J*(H,H) = 6.3 Hz, 1 H), 5.62 (d, *J*(H,H) = 6.3 Hz, 1 H), 5.84 (br. s, 1 OH), 7.28–7.39 (m, 1 H), 7.47–7.86 (m, 9 H-Ar), 8.06–8.21 (m, 2 H) ppm; ^{19}F NMR (188.3 MHz, d_6-DMSO, r.t.): δ = –53.8 (s), –54.0 (br. s), –57.4 (s) ppm; IR (KBr): ν = 3509m, 2995w, 1713m, 1448m, 1387m, 1314s, 1159s, 1136s, 1066m, 1038m, 880w, 842w, 765m, 664m cm^{-1}; MS (ESI, NH_4OAc/MeOH): neg. mode: *m/z* = 591.3 $[M–H]^-$; pos. mode: *m/z* = 610.3 $[M+NH_4]^+$.

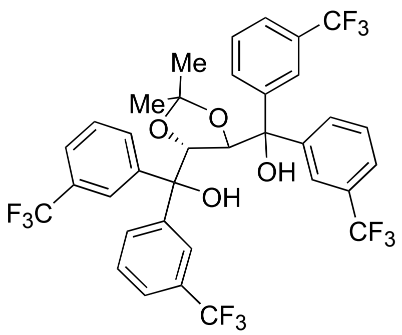

T6: **((4*R*,5*R*)-2,2-Dimethyl-1,3-dioxolane-4,5-diyl)bis(bis(3-(trifluoromethyl)phenyl)methanol) (T6)**. To a Grignard solution obtained from Mg-turnings (972 mg, 40 mmol), THF (20 mL) and 3-bromo-benzotrifluoride (5.11 mL, 37 mmol), dimethyl (*R*,*R*)-2,3-di-*O*-isopropylidene-tartrate (**7**) (1.614 g, 7.4 mmol) was added dropwise under stirring with cooling in a water bath at r.t. The reaction mixture was stirred. overnight, quenched with NH_4Cl aq and *t*-BuOMe. The organic phase was washed with NH_4Cl aq and evaporated. The residue was purified by CC (*t*-BuOMe/hexane 1:10+3% NEt_3→1:2+3% NEt_3→1:2+3% iPrOH). The combined product fractions were evaporated and the residue dissolved in toluene. NEt_3 was removed by washing with HCl (0.3 M). Evaporation and drying in HV for 1 d at 50 °C gave a solid. After pulverization and drying (1 d, 0.002 mbar, 60 °C), a faint yellow powder (4.406 g, 81%) was obtained.

R_f = 0.1 (*t*-BuOMe/hexane 1:10); m.p. 89–91 °C; $[\alpha]_D$ = –35.1 (c = 1.26 in MeOH); ^{1}H NMR (300 MHz): δ = 1.07 (s, 6 H), 4.18 (s, 2 H; OH), 4.44 (s, 2 H), 7.35–7.44 (m, 2 H), 7.46–7.56 (m, 6 H), 7.58–7.66 (m, 4 H), 7.73–7.80 (m, 4 H) ppm; ^{13}C NMR (75.5 MHz): δ = 26.8, 77.7, 80. 9, 110.2, 123.9 (q, *J*(F,C) = 273 Hz), 124.1 (q, *J*(F,C) = 273 Hz), 124.1 (q, *J*(F,C) = 4 Hz), 124.8 (q, *J*(F,C) = 4 Hz), 125.0 (q, *J*(F,C) = 4 Hz), 125.4 (q, *J*(F,C) = 4 Hz), 128.3, 129.0, 130.2 (q, *J*(F,C) = 50 Hz), 130.7 (q, *J*(F,C) = 50 Hz), 130.8 (q, *J*(F,C) = 1 Hz), 131.3 (q, *J*(F,C) = 1 Hz), 142.6, 145.5 ppm; ^{19}F NMR (188.3 MHz): δ = –62.9 (s), –63.0 (s) ppm; IR (KBr): ν = 3600w, 3347m (br.), 3220sh, 2990w, 2892w, 1965w, 1907w, 1616w, 1492w, 1436m, 1384m, 1376m, 1330s,

1169s, 1125s, 1077s, 886m, 805m, 707s cm^{-1}; MS (EI): *m/z* (%) = 719 (10) [M–F]$^+$, 663 (3), 643 (2), 419 (4), 403 (9), 373 (64), 319 (85), 303 (24), 173 (100), 145 (35), 101 (17); analysis calcd (%) for $C_{35}H_{26}F_{12}O_4$ (738.57): C 56.92, H 3.55; found C 56.78, H 3.75.

The substance forms a strongly hydrogen-bonded solvate with NEt_3. Selected ^{1}H NMR-data of the solvate (250 MHz): δ = 0.83 (t, ^3J(H,H) = 7.2 Hz, 9 H; NCH_2*Me*), 1.12 (s, 6 H; 2 Me), 2.02–2.35 (m, 6 H; NCH_2Me), 4.36 (s, 2 H; CH), 9.04 (br. s, 2 H; OH) ppm.

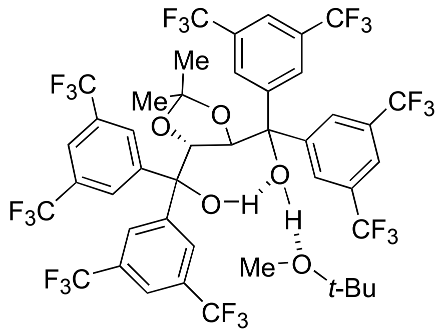

T7: (4*R*-*trans*)-2,2-Dimethyl-α,α,α',α'-tetra{bis(3,5-trifluoromethyl)phenyl}-1,3-dioxolane-4,5-dimethanol, *t*-BuOMe-solvate (T7·*t*-BuOMe) [9]. A suspension of Mg turnings (760 mg, 31.3 mmol) in THF (15 mL) with two small crystals of iodine was heated to 40 °C. A small portion (ca. 0.5 mL) of 3,5-bis-trifluoromethyl-bromobenzene (out of 5.1 mL, 29.8 mmol) was added and the mixture stirred until the Grignard-reaction started. The remaining aryl bromide was slowly added under cooling of the reaction vessel in a r.t. water bath. After completion of the addition, the mixture was stirred for 1 h at 50 °C. After cooling in an ice bath, the Grignard reagent turned into a suspension. The ice bath was removed and dimethyl (*R*,*R*)-2,3-di-*O*-isopropyliden tartrate (1.10 mL, 6.0 mmol) was added dropwise within 15 min to give a clear dark-green solution with warming. After 2 d of stirring at r.t. (actual reaction time may be considerably shorter) the reaction mixture was carefully quenched by addition of sat. aq NH_4Cl (20 mL), 2 M HCl aq (5 mL), water (100 mL) and *t*-BuOMe (100 mL), and the organic phase washed with sat. aq NH_4Cl (3×). The crude was purified by CC (*t*-BuOMe/hexanes 1:30–1:20) to give a yellow resin that was dried over 3 d at 60 °C in HV. After cooling to r.t., the resin was pulverized with a spatula, and the yellowish powder dried for another 2 d in HV. According to ^{1}H NMR and elemental analysis, the product was a solvate with one equivalent of *t*-BuOMe per TADDOL unit. Yield: 5.405 g (82%). The powder sintered to a yellow resin within a few days.

R_f = 0.36 (*t*-BuOMe/hexanes 1:10); $[\alpha]_D$ = –26.6 (c = 1.15, CH_2Cl_2; Lit. value [9] = –23.3 (*c* = 1.0, $CHCl_3$); ^{1}H NMR (300 MHz): δ = 1.01 (s, 9 H, *t*-BuOMe), 1.12 (s, 6 H, 2 Me), 2.80 (s, 3 H, *t*-BuOMe), 4.19 (s, 2 H, CH), 6.11 (s, 2 OH), 7.81 (s, 4 H_{Ar}), 7.84 (s, 2 H_{Ar}), 7.95 (s, 2 H_{Ar}), 8.12 (s, 4 H_{Ar}) ppm; ^{13}C NMR (75.5 MHz): δ = 26.5, 26.5, 48.6, 76.7, 81.3, 11 0.7, 122.6 (m), 123.0 (q, *J*(F,C) = 273 Hz), 123.1 (q, J(F,C) = 273 Hz), 127.6 (m), 128.3 (m), 131.8 (sept, *J*(F,C) = 34 Hz), 143.4, 145.8 ppm; ^{19}F NMR (188.3 MHz): δ = –63.4 (s, CF_3), –63.5 (s, CF_3) ppm; IR (KBr): ν = 3606w, 3410m (br.), 3109w, 2992w, 2942w, 2898w, 1624w, 1468w, 1375s, 1279s, 1174s, 1132s, 900m, 846m, 711m, 683s cm^{-1}; analysis calcd (%) for $C_{39}H_{22}F_{24}O_4$+$C_5H_{12}O$ (1098.72): C 76.26, H

9.89; found C 76.05, H 10.02. N otes: This compound was previously obtained by a similar procedure in 24% yield as a solvent-free material [9]. Several hints from the ^{1}H NMR imply that our product is a strongly hydrogen-bonded solvate with a molecule of *t*-BuOMe: The OH signal of the solvent-free material appears at $\delta(^{1}H)$ = 4.66 ppm [9], but is shifted to δ = 6.11 ppm in the solvate; furthermore, aromatic deshielding in the solvate causes a low-frequency shift of the *t*-BuOMe signals, relative to the free solvent. The removal of the trace amounts of solvents is difficult, but was not necessary for our purposes.

T8: (4*R*-*trans*)-2-Methyl-2-phenyl-α,α,α',α'-tetraphenyl-1,3-dioxolan-4,5-dimethanol: Material obtained by courtesy of Albert K. Beck (Seebach group, ETH Zürich).

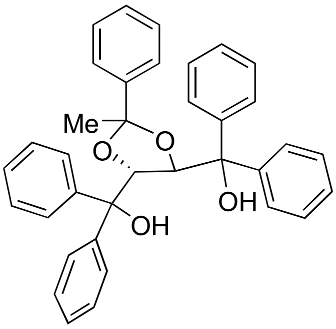

T9: (4*R*-*trans*)-2-*tert*-Butyl-2-methyl-α,α,α',α'-tetra-(1-naphthyl)-1,3-dioxolan-4,5-dimethanol: Material obtained by courtesy of Albert K. Beck (Seebach group, ETH Zürich).

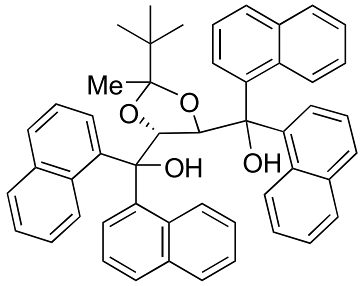

T10: (4*R*-*trans*)-2,2-Dimethyl-α,α,α',α'-tetra-(1-methoxyphenyl)-1,3-dioxolan-4,5-dimethanol: Material obtained by courtesy of Albert K. Beck (Seebach group, ETH Zürich).

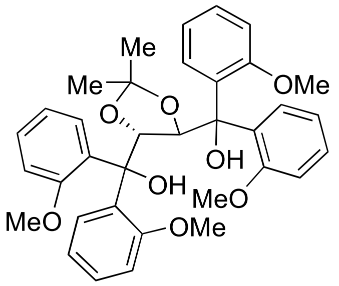

6. Ligand-screening experiments

Dichloro-diisopropoxy-titanium(IV): The complex [$TiCl_2(OiPr)_2$] was prepared following a literature procedure [10]: To a solution of Ti(O*i*Pr)$_4$ (6.0 mL, 20.4 mmol) in dry *n*-hexane (20 mL), $TiCl_4$ (2.2 mL, 20.0 mmol) was added dropwise under stirring and cooling in a r.t. water bath. From the initially clear, colorless solution, crystals of the product soon started to separate. Filtration under argon, washing of the solid with dry pentane (5 mL) and drying in HV gave white crystals of [$TiCl_2(OiPr)_2$] (5.566 g, 59%).

^{1}H NMR (300 MHz): δ=1.44 (d, J = 6.2, 12 H, Me), 4.86 (sept, J = 6.2, 2 H, $CHMe_2$) ppm;

^{13}C NMR (75.5 MHz): δ= 25.4 (CH_3), 87.4 (br., CH) ppm.

General procedures for preparing in situ $TiCl_2$(DIOLate) catalysts:

• ***Variation 1: Stock solution for multiple catalyses***: A solution of [TiCl$_2$(O*i*Pr)$_2$] (1 M in MeCN; 0.62 mL, 0.62 mmol) was added to a stirred solution (or: suspension) of the ligand (0.63 mmol) in dry MeCN (9 mL). Stirring was continued for 1 d at r.t., and the solution used as such. For a single catalytic reaction (0.25 mmol scale), 0.20 mL (ca. 0.062 M) of this solution was used.

• ***Variation 2: Small-scale, in situ, catalyst preparation***: In a glove-box, solid [TiCl$_2$(O*i*Pr)$_2$] (12 mg, 0.05 mmol) and ligand (0.06 mmol) were combined with MeCN (1 mL). After being stirred for 1 d at r.t., a quarter (ca 0.25 mL) of this solution was injected into a single catalytic reaction (0.25 mmol scale). In case of **T4** , THF was added to the in situ catalyst preparation, for solubility reasons. The in situ catalyst mixture was evaporated to dryness and the solid residue dissolved in THF for addition to the catalytic reaction in MeCN.

Substrates and products

See the subsequent paper for synthesis and characterization of those products [1].

Benzyl 2-methyl-3-oxo-pentanoate (10):[11] See the subsequent paper for synthesis and characterization.

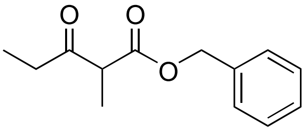

Benzyl 2-fluoro-2-methyl-3-oxo-pentanoate (**10-F**): See the subsequent paper for characterization and chiral analytics (HPLC).

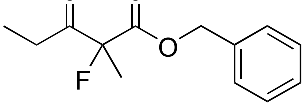

2’,4’,6’-Triisopropylbenzyl 2-methyl-3-oxo-pentanoate (11): See the subsequent paper for synthesis and characterization.

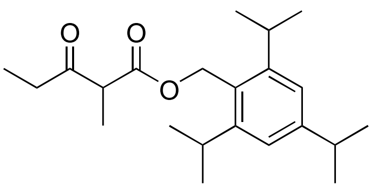

2’,4’,6’-Triisopropylbenzyl 2-fluoro-2-methyl-3-oxo-pentanoate (11-F): See the subsequent paper for characterization and chiral analytics (HPLC).

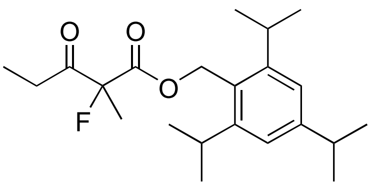

7. Stereochemical-correlation experiments

Correlation of 11-F with 10-F

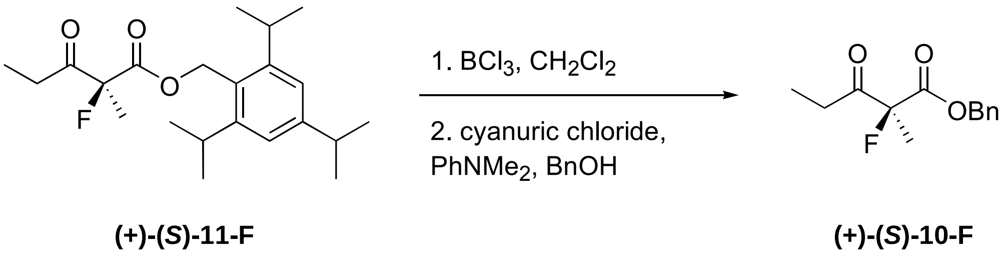

Benzyl 2-fluoro-2-methyl-pentanoate (10-F): A solution of (+)-**11-F** (98 mg, 0.27 mmol; 85.6% ee) in CH_2Cl_2 (2 mL), containing powdered molecular sieves 3 Å (200 mg), was cooled to –20 °C (MeOH/ice). A solution of BCl_3 (1 M in hexane, 0.30 mL, 0.30 mmol) was added dropwise under stirring. After 30 min, TLC indicated that all starting material had been consumed. While still at –20 °C, benzyl alcohol (1 mL, 10 mmol) and cyanuric chloride (188 mg, 1 mmol) were added to the reaction mixture, followed by dropwise addition with stirring of *N*,*N*-dimethylaniline (0.65 mL, 5 mmol). The cooling bath was removed and the reaction mixture stirred for 2 d at r.t., followed by workup with *t*-BuOMe/H_2O. The organic phase was washed with sat. aq NaCl and filtered over neutral Al_2O_3. After evaporation, the residue was separated by CC (SiO_2, *t*-BuOMe/hexanes 1:20) to give the product (50 mg, 77%). Identification of this substance as **10-F** was assured by comparison of TLC, ^{1}H NMR and chiral HPLC data (84.9 ee). The measured ee value is sufficiently close to the ee of the starting material used (**11-F**, 85.6 ee), within the limits of accuracy of the analytical methods used.

HPLC (Chiralcel OJ, hexane/iPrOH 96:4, 1 mL/min): t_R = 19.0 min (∫ = 7.54%), 21.4 min (∫ = 92.46%).

Correlation of 10-F with 12

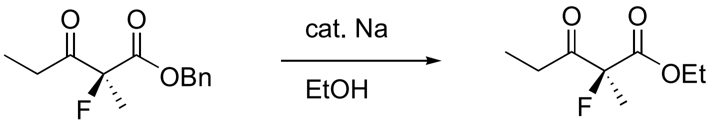

(+)-(*S*)-10-F **(+)-(*S*)-12**

Ethyl 2-fluoro-2-methyl-3-oxopentanoate (12). To a stirred solution of (+)-(*S*)-**10-F** (132 mg, 0.55 mmol; 67–69% ee by HPLC; value of lower accuracy because of minor impurity peaks) in EtOH (99.9%; 2.0 mL), finely ground molecular sieves 3Å (300 mg) were added and the solution was stirred for 1 h at r.t. After addition of a small piece of metallic sodium (ca 1.5 mg, 0.065 mmol) the mixture was stirred at r.t. According to TLC after 10 min, a strong spot for benzyl alcohol had developed, whereas the spot for the remaining starting material was very weak. The product was hardly detected by UV or staining agents ($KMnO_4$ *aq*, Mostain, dinitrophenylhydrazine). The reaction mixture was diluted with more EtOH (2.0 mL). After being stirred for another 15 min, Amberlyst ion-exchange resin (H^+-form, washed with EtOH, 150 mg) was added and the mixture stirred for 10 min. Filtration through Celite, washing of the filter with *t*-BuOMe and careful evaporation of the filtrate in a rotatory evaporator at 100 mbar/50 °C to a small volume gave the crude, which was dried for 5 min at 50 mbar/50 °C. The fruity-smelling residue was dissolved in MeOH (2.0 mL) and the optical rotation recorded as α = +1.428° (MeOH). Assuming quantitative conversion, the crude material would have contained 0.55 mmol = 96.9 mg of product, thus $[\alpha]_D$ = +29.47 (c = 4.845, MeOH), corresponding to an optical purity of 55% (using the literature value of $[\alpha]_D$ = +53.5 {c = 1.19, MeOH}) [12]. The discrepancy to the ee value of the starting material (68% ee) is probably due to losses of the volatile product on evaporation.

Next, the MeOH-solution was again carefully evaporated in order to record NMR spectra of the residue. The expected ester **12** was identified as mixture with benzyl alcohol, EtOH, MeOH and a small quantity (5%) of starting material **10-F**. After CC (SiO_2, *t*-BuOMe/hexane 1:5) and pooling of the product fractions, which were identified by their fruity smell, careful evaporation and drying (10 min at 60 mbar/50 °C) gave 64 mg of a colorless, fruity smelling liquid. This consisted (^{1}H NMR analysis) of 84 mol % of **12**, 10% of *n*-hexane, 2% of *t*-BuOMe, and 4% of **10-F**. A product yield of 55% can be calculated for **12**.

Data for **12**: $[\alpha]_D$ = +33.9 (*c* = 1.25[a], MeOH); ^{1}H NMR (250 MHz): δ = 1.09 (t, *J* = 7.2 Hz, 3 H, $MeCH_2$), 1.30 (t, *J* = 7.1 Hz, 3 H, $MeCH_2O$), 1.69 (d, *J* = 22.2 Hz, MeCF), 2.65 (dqd, *J* = 19.0, 7.3, *J*(F,H) = 2.7 Hz, 1 H, C*H*$_2$Me; A of ABM_3X system), 2.76 (dqd, *J* = 19.0, 7.2, *J*(F,H) = 3.4 Hz, 1 H, C*H*$_2$Me; B of ABM_3X system), 4.27 (q, *J* = 7.1 Hz, 2 H, OC*H*$_2$Me) ppm; ^{13}C NMR (62.9 MHz): δ =

7.0 (d, J(F,C) = 2 Hz, CH_3), 13.9 (CH_3), 20.1 (d, J(F,C) = 23 Hz, CH_3), 30.5 (CH_2), 62.5 (CH_2), 97.7 (d, J(F,C) = 194 Hz, CF), 167.0 (d, J(F,C) = 25 Hz, C=O), 205.1 (d, J(F,C) = 27 Hz, C=O). GC: (*Supelco* ®-dex column, 30 m × 0.25 mm × 0.25 µm; 82 °C isothermal; He constant flow = 1.4 mL/min; FID-detection): t_R = 15.1 min (*R*-**12**), 16.3 min (*S*-**12**).

Notes: [a] Uncorrected specific rotation of the impure sample. The following corrections can be applied: the purity of the product is ca 84% (^{1}H NMR) and it contains 4 % of **10-F** as an optically active impurity ($[\alpha]_D$ = +32.5 {c = 0.905, MeOH; @ 70.8 ee}). Taking into account those corrections, comparison with the literature value (+53.5; see above) indicates an optical purity of ca. 73% (*S*) for the ethyl ester **12**. This value is sufficiently close to the 67–69% ee of the starting material, taking into account experimental errors.

Correlation of (+)-6-F (and other 2-fluoro-2-methylpropanoates) with (+)-(*S*)-12:

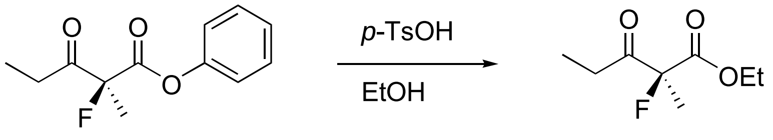

(+)-(*S*)-6 **(+)-(*S*)-12**

A solution of (+)-**6-F** (22 mg, 0.1 mmol) and *p*-TsOH (17 mg, 0.1 mmol) in EtOH (99.9%; 0.25 mL) was stirred for 1 d at r.t. A sample for chiral GC-analysis was directly removed. Correlation with the known retention times for **12** (see above) indicated the same excess (*S*)-configuration, which was also found in samples of **12** obtained as above from **10-F**.

8. Mechanistic experiments

Ionization of [TiCl$_2$(TADDOLate)(dme)] with AgBF$_4$

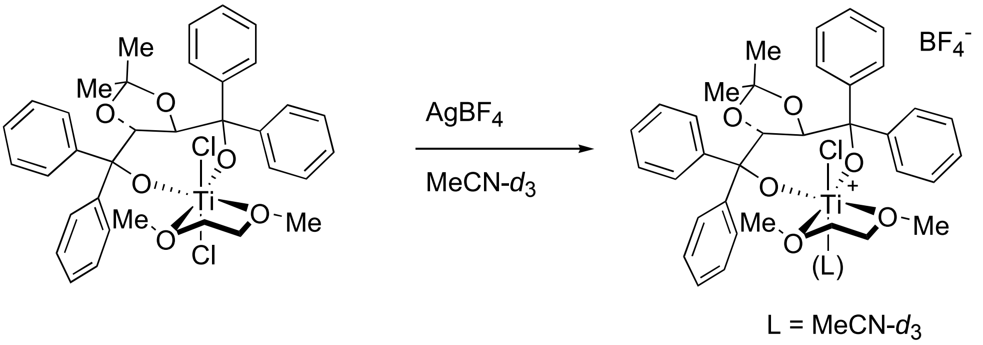

To a solution of **K1** (5.0 mg, 7.1 µ mol) in MeCN-d_3 (0.8 mL) was added AgBF$_4$ (1.4 mg, 7.2 µmol). The mixture was shaken and set aside overnight, forming a grey precipitate. The supernatant solution was used for recording an ^{1}H NMR spectrum.

^{1}H NMR (250 MHz, CD$_3$CN): δ = 0.48 (s, 3 H, CH$_3$), 0.93 (s, 3 H, CH$_3$), 3.31 (br s, 6 H, 2 OCH$_3$ of dme), 3.48 (s, 4 H, OCH$_2$CH$_2$O of dme), 5.08 (d, J = 7.2 Hz, 1 CH dioxolane), 5.20 (d, J = 7.2 Hz, 1 CH dioxolane), 7.20–7.68 (m, 20 H$_{Ph}$) ppm.

Ionization of [TiCl$_2$(TADDOLate)(dme)] in the presence of F-TEDA

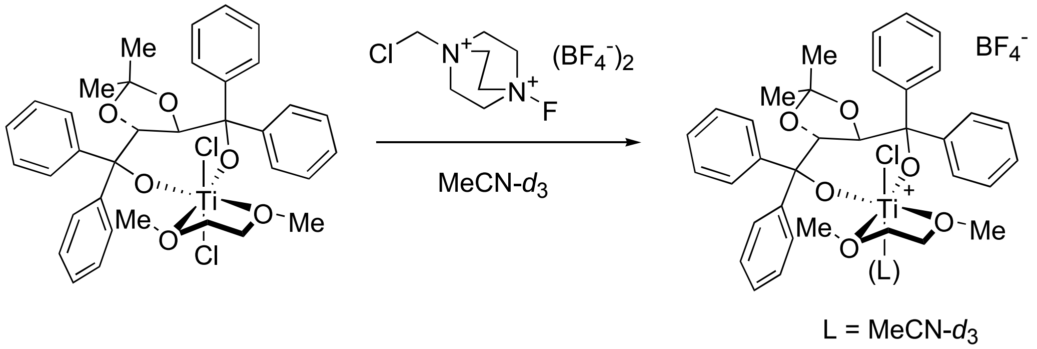

A suspension of **K1** (7.1 mg, 10 µmol) and F-TEDA (177 mg, 0.50 mmol) was stirred in MeCN-d_3 (1 mL) for 1.5 h. The mixture was filtered into an NMR-tube. The solid residue was extracted with CDCl$_3$, but contained almost no soluble material (by ^{1}H NMR). The filtrate gave the following spectrum:

^{1}H NMR (250 MHz, CD$_3$CN): δ = 0.64 (s, 3 H, Me), 1.01 (s, 3 H, Me), 3.37 (br s, 6 H, 2 OMe of dme), 3.54 (br s, 4 H, OCH$_2$CH$_2$O of dme), 5.25 (br s, 1 CH of dioxolane), 5.64 (br s, 1 CH of dioxolane), 7.19–7.63 (m, 20 H$_{Ph}$) ppm. Signals for F-TEDA: δ = 4.23–4.38 (m, 6 H), 4.66–

4.84 (m, 6 H), 5.32 (s, 2 H) ppm. Additional signals for $[ClCH_2\{N(CH_2CH_2)_3N\}H](BF_4)_2$: δ = 3.70–3.95 (m, 12 H), 5.21 (s, 2 H) ppm.

References

[1] A. Bertogg, L. Hintermann, D. P. Huber, M. Perseghini, M. Sanna, A. Togni, *Helv. Chim. Acta,* in press. doi:10.1002/hlca.201100375

[2] R. E. Banks, M. K. Besheesh, S. N. Mohialdin-Khaffaf, I. Sharif, *J. Chem. Soc., Perkin Trans. 1* **1996**, 2069.

[3] L. Hintermann, D. Broggini, A. Togni, *Helv. Chim. Acta* **2002**, *85*, 1597.

[4] E. C. Horning and A. F. Finelli, *Organic Syntheses* **1963**, *Coll. Vol. 4*, 461.

[5] R. E. Banks, N. J. Lawrence, A. L. Popplewell, *J. Chem. Soc., Chem. Commun.* **1994**, 343.

[6] A. Nudelman, R. Kelner, N. Broida, H. E. Gottlieb, *Synthesis* **1989**, 387.

[7] A. K. Beck, B. Bastani, D. A. Plattner, W. Petter, D. Seebach, H. Braunschweiger, P. Gysi, L. La Vecchia, *Chimia* **1991**, *45*, 238.

[8] D. Seebach, A. K. Beck, A. Heckel, *Angew. Chem. Int. Ed.* **2001**, *40*, 92.

[9] a) R. Dahinden, *Ti-TADDOLat vermittelte Cycloadditionen und Reduktionen von aromatischen Ketonen durch modifizierte TADDOL-LAH-Reagenzien*, ETH Dissertation 11822, ETH Zürich, 1996. b) D. Seebach, A. K. Beck, R. Dahinden, M. Hoffmann, F. N. M. Kuehnle, *Croatica Chemica Acta* **1996**, *69*, 459.

[10] K. Mikami, M. Terada, T. Nakai, *J. Am. Chem. Soc.* **1990**, *112*, 3949.

[11] B. Loubinoux, J.-L. Sinnes, A. C. O'Sullivan, *J. Chem. Soc., Perkin Trans 1* **1993**, 405.

[12] T. Kitazume, T. Kobayashi, *J. Fluorine Chem.* **1986**, *31*, 357.
